# TRPA1–FGFR2 binding event is a regulatory oncogenic driver modulated by miRNA-142-3p

**DOI:** 10.1038/s41467-017-00983-w

**Published:** 2017-10-16

**Authors:** Jonathan Berrout, Eleni Kyriakopoulou, Lavanya Moparthi, Alexandra S. Hogea, Liza Berrout, Cristina Ivan, Mihaela Lorger, John Boyle, Chris Peers, Stephen Muench, Jacobo Elies Gomez, Xin Hu, Carolyn Hurst, Thomas Hall, Sujanitha Umamaheswaran, Laura Wesley, Mihai Gagea, Michael Shires, Iain Manfield, Margaret A. Knowles, Simon Davies, Klaus Suhling, Yurema Teijeiro Gonzalez, Neil Carragher, Kenneth Macleod, N. Joan Abbott, George A. Calin, Nikita Gamper, Peter M. Zygmunt, Zahra Timsah

**Affiliations:** 1 School of Molecular and Cellular Biology, University of Leeds, Leeds, LS2 9JT UK; 2 Division of Clinical Chemistry and Pharmacology, Lund University, 221 85 Lund, Sweden; 3 School of Biomedical Sciences, University of Leeds, Leeds, LS2 9JT UK; 4 The University of Texas at Brownsville, Brownsville, TX 78520 USA; 5 Department of Gynecologic Oncology and Reproductive Medicine, The University of Texas MD Anderson Cancer Center, Houston, TX 77054 USA; 6 Leeds Institute of Cancer and Pathology, Wellcome Trust Brenner Building, St James’s University Hospital, LS9 7TF Leeds, UK; 7 Faculty of Medicine and Health, Leeds Institute of Cardiovascular and Metabolic Medicine, University of Leeds, Leeds, LS2 9JT UK; 8 Department of Genomic Medicine, The University of Texas MD Anderson Cancer Center, Houston, TX 77054 USA; 9 Faculty of Medicine and Health, Leeds Institute of Cancer and Pathology, University of Leeds, Leeds, LS2 9JT UK; 10 Centre for Biotechnology, Anna University, Chennai, Tamil Nadu 600025 India; 11 Department of Veterinary Medicine and Surgery, The University of Texas MD Anderson Cancer Center, Unit 63, Houston, TX 77054 USA; 12 Section of Pathology and Tumor Biology, Leeds Institute of Cancer and Pathology, Wellcome Trust Brenner Building, St James’s University Hospital, Leeds, LS9 7TF UK; 13 Department of Physics, King’s College London, Strand, London WC2R 2LS UK; 14 Cancer Research UK Edinburgh Centre, Institute of Genetics and Molecular Medicine, University of Edinburgh, Edinburgh, EH4 2XR UK; 15 Institute of Pharmaceutical Science, Franklin-Wilkins Building, King’s College London, London, SE1 9NH UK; 16 Department of Experimental Therapeutics, Division of Cancer Medicine, The University of Texas MD Anderson Cancer Center, Houston, TX 77054 USA

## Abstract

Recent evidence suggests that the ion channel TRPA1 is implicated in lung adenocarcinoma (LUAD), where its role and mechanism of action remain unknown. We have previously established that the membrane receptor FGFR2 drives LUAD progression through aberrant protein–protein interactions mediated via its C-terminal proline-rich motif. Here we report that the N-terminal ankyrin repeats of TRPA1 directly bind to the C-terminal proline-rich motif of FGFR2 inducing the constitutive activation of the receptor, thereby prompting LUAD progression and metastasis. Furthermore, we show that upon metastasis to the brain, TRPA1 gets depleted, an effect triggered by the transfer of TRPA1-targeting exosomal microRNA (miRNA-142-3p) from brain astrocytes to cancer cells. This downregulation, in turn, inhibits TRPA1-mediated activation of FGFR2, hindering the metastatic process. Our study reveals a direct binding event and characterizes the role of TRPA1 ankyrin repeats in regulating FGFR2-driven oncogenic process; a mechanism that is hindered by miRNA-142-3p.

## Abstract (web summary)

TRPA1 has been reported to contribute lung cancer adenocarcinoma (LUAD), but the mechanisms are unclear. Here the authors propose that TRPA1/FGFR2 interaction is functional in LUAD and show that astrocytes oppose brain metastasis by mediating the downregulation of TRPA1 through exosome-delivered miRNA-142-3p.

## Introduction

Lung cancer is the leading cause of cancer-related mortality and the second most common type of cancer worldwide^1^. Lung adenocarcinoma (LUAD) accounts for 40% of all lung cancer cases; it often metastasizes to the liver, adrenal glands, bones, and brain^2, 3^. Notably, ~50% of all cases of brain metastases originate from lung cancer, where early metastatic spread to the brain is hard to detect, and thus long-term survival of patients is very rare^4–6^. The role of the brain metastatic niche in regulating tumor progression remains controversial. Some studies have shown that brain astrocytes support the survival of cancer cells in a dormant state, by inhibiting further proliferation and invasion, while others describe a mechanism that supports the metastatic process^7, 8^.

Recently, it has been reported that the ion channel, transient receptor potential ankyrin-1 (TRPA1), which is expressed in nociceptive neurons and acts as a chemosensor of noxious compounds, is implicated in lung malignancies^9–12^. While TRPA1 has been shown to be expressed in non-neuronal cells as well (e.g., lung epithelial fibroblasts), little is known about its function outside the somatosensory system, even less in malignancies^11–13^. TRPA1 possesses an extended C-terminal domain, which is important for subunit interactions during channel assembly. Its N-terminal region contains 16 ankyrin repeats with a putative, yet uncharacterized, role in pore-gating and mediating protein–protein interactions, where the binding partners are yet-to-be identified^11, 14^. Interestingly, a regulatory protein–protein interaction has been reported to occur between the ankyrin repeats of ANKRA protein and the proline-rich cytoplasmic domain of megalin receptor^15^. This prompted us to investigate the regulatory role of TRPA1 ankyrin repeats in LUAD.

In lung malignancies, and specifically LUAD, we have previously shown that the membrane receptor, fibroblast growth factor receptor 2 (FGFR2), is a critical driver of disease progression, especially under non-stimulated conditions^16–19^. In this case, FGFR2 recruits proteins to its C-terminal proline-rich motif to trigger signaling cascades and aberrant cellular functions independent of extracellular stimulation^17^.

All of the above urged us to investigate the potential interaction between TRPA1 and FGFR2 in LUAD. In the present study, we reveal a direct binding event between ankyrins 6–10 of TRPA1 and prolines 810–813 of FGFR2, which constitutively activates the receptor and its signaling pathways independent of extracellular stimulation. TRPA1-FGFR2 supports the oncogenic process in LUAD and its metastasis to the brain. Our study also uncovers that upon encounter with astrocytes in the brain, LUAD cells are depleted of TRPA1, which inhibits FGFR2- driven cellular proliferation and invasion. We demonstrate that this occurs by the transfer of TRPA1-targeting exosomal miRNA-142-3p from astrocytes to LUAD (as illustrated in Supplementary Fig. 1).

## Results

### C-terminal region of FGFR2 binds to TRPA1 ankyrin repeats

**Fig. 1:**
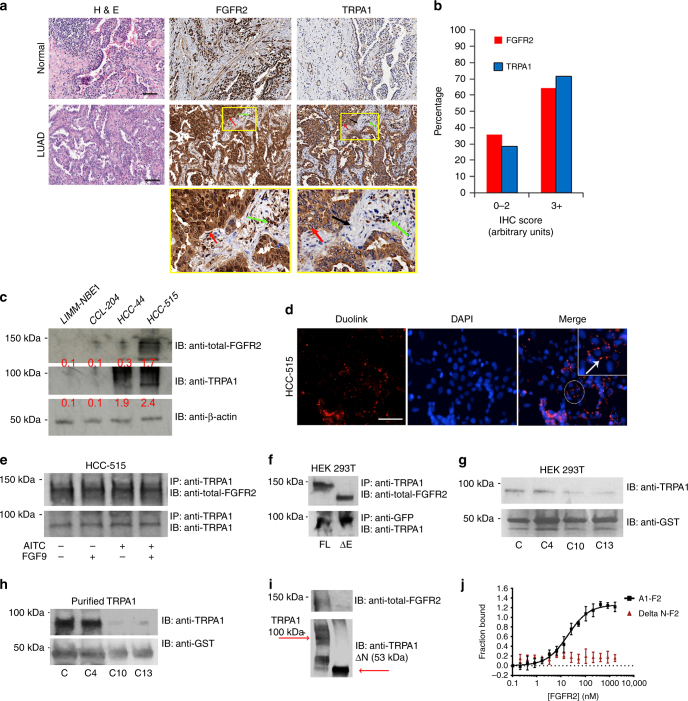
FGFR2 binds directly to TRPA1 ankyrin repeats via its C-terminal proline-rich motif. **a** Representative image of haematoxylin and eosin (H&E) stain and immunohistochemistry (IHC) staining with FGFR2 and TRPA1 antibodies of a lung cancer tissue microarray. Scale bar: 10 μm. Neoplastic epithelial cells are indicated by the red arrow, inflammatory cells by the green arrow, and supporting stroma (fibroblasts) by the black arrow. **b** Fraction of total LUAD samples (in percentage) in the tissue microarrays (TMAs) with the indicated pathological scores. **c** Western blots for the expression levels of FGFR2 and TRPA1 in LIMM-NBE1, CCL-204, HCC-515, and HCC-44 cell lines. Normalized densitometric values (in relative arbitrary units) are reported in red below each band and represent the average of three independent experiments. **d** Duolink assay results with an example of a PLA signal depicted by the white arrow. Scale bar: 50 μm. **e** Western blot analysis of an immunoprecipitation (IP with anti-TRPA1) in HCC-515 cell line followed by immunoblotting (IB) with the indicated antibodies. **f** Western blot analysis of an IP in HEK 293T cells transfected with full-length TRPA1 with either full-length FGFR2 (FL) or extracellular region-truncated FGFR2 (ΔE). IP and IB were performed with the indicated antibodies. **g** GST pull-down results in HEK 293T cells transfected with TRPA1 and C, C4, C10, and C13 GST-constructs on GST beads. **h** GST pull-down experiment with full-length purified TRPA1 and C, C4, C10, and C13 GST-constructs on GST beads. **i** Western blot analysis of purified biotinylated full-length TRPA1 or ankyrin repeats-truncated TRPA1 (ΔN) proteins immobilized on streptavidin beads and incubated with full-length purified FGFR2. **j** Microscale thermophoresis (MST) curve of purified full-length FGFR2 (F2; titrated in a range of concentrations) with purified full-length TRPA1 (A1) or ΔN. Apparent dissociation constant *K*_d_ for A1-F2 was 122.8 ± 23.4 nM. Error bars represent the s.d. of each data point (*n* = 7)

We assessed the expression level of both the proteins in LUAD by performing an immunohistochemical (IHC) analysis of a tissue microarray containing 102 normal and lung cancer tissue samples (Fig. 1a, b). Unlike in normal tissues, it is evident that both the proteins are highly expressed in LUAD samples with a pathological score of 3^+^ in 60–70% of the cancer tissues investigated (Fig. 1b). Compared to normal tissues (as shown in the zoomed-in yellow boxes), neoplastic epithelial cells in LUAD samples stained strongly positive for FGFR2 (red arrow). Most of the stroma is negative for FGFR2 staining, but the inflammatory cells infiltrated into the stroma have positive FGFR2 staining (green arrow). For TRPA1, there is a strong positive staining of the neoplastic epithelial cells (red arrows). The supporting stroma (fibroblasts) is negative for TRPA1 staining (black arrow), and contains variable numbers of infiltrated inflammatory cells that stain positive for TRPA1 (green arrow) (Fig. 1a).

To investigate the expression level of both the proteins at a cellular level, we employed a panel of LUAD cell lines (HCC-515, HCC-44, HCC-827, and NCI-H1793) along with the normal lung cell line (CCL-204) in immunofluorescence (IF) staining (Supplementary Fig. 2a, b). The results show that the expression level of these proteins in LUAD is cell-type specific; both the proteins are highly expressed in HCC-515 cell line, but only TRPA1 was detected in HCC-44. Expression level of both the proteins (especially TRPA1) is low in HCC-827 and NCI-H1793, and hence both cell lines were not used for further investigation in this study. Western blot results in Fig. 1c show that TRPA1 is expressed in LUAD cell lines (HCC-515 and HCC-44), whereas FGFR2 is only detectable in HCC-515 compared to CCL-204 and normal bronchial epithelium cell line (LIMM-NBE1; used as an additional control).

To test for potential co-localization of TRPA1 and FGFR2, we performed a Duolink proximity ligation assay (PLA) in HCC-515 cells using antibodies that are targeted against endogenous FGFR2 and TRPA1 (Fig. 1d). In this technique, two proteins within a sample are labeled with specific primary antibodies followed by recognition with secondary antibodies conjugated with short DNA strands. If the two proteins are co-localized within less than ~ 40 nm of each other, the two DNA probes are ligated, a unique new DNA sequence is amplified, and a color reaction is developed, resulting in characteristic PLA punctate staining pattern. Thus, this technique relies on both antibody recognition and DNA amplification, making it more specific and sensitive than other microscopy-based techniques^20^. The presence of distinct PLA puncta (white arrow pointing at red fluorescent dots) in HCC-515 cells indicates that the two proteins may indeed co-localize to form a complex.

Next, we performed an immunoprecipitation (IP) experiment in HCC-515 cells and HCC-44 cells (Fig. 1e and Supplementary Fig. 2c–e). In HCC-515 (upper panel), TRPA1-FGFR2 complex formation was constitutive and prevailed under non-stimulated conditions, and following stimulation with fibroblast growth factor 9 (FGF9; FGFR2 agonist) and/or Allyl isothiocyanate (AITC; TRPA1 agonist). In HCC-44 cells (Supplementary Fig. 2d, e), IP with anti-TRPA1 pulled down a minimal amount of FGFR2 relative to HCC-515 cells, especially if normalized to the control lane (IP and IB with anti-TRPA1). Also, an IP with anti-FGFR2 in HCC-44 cells failed to pull down TRPA1.

Similar results were obtained in HEK 293T cells (Supplementary Fig. 2f, g) that was utilized as our model cell line, since they are easy to transfect and lack the expression of both the proteins. Cells were transfected with full-length FGFR2 and TRPA1-GFP (Supplementary Fig. 2f) followed by IP (Supplementary Fig. 2g).

To further test whether this binding event depends on FGFR2 stimulation, we co-transfected HEK 293T cells with TRPA1-GFP and the truncated version of FGFR2 (ΔE, which lacks the extracellular FGF9-binding region). IP results in Fig. 1f indicate that TRPA1 can successfully form a complex with both full-length (FL) FGFR2 and its truncated version. To test whether FGFR2 intracellular prolines (P810–P813, known to promote protein–protein interactions in the absence of extracellular stimulation^17–19^) are responsible for TRPA1-FGFR2 complex formation, we fused the last 58 amino acids of the receptor (residues 764–821) with Glutathione S-transferase (GST) to generate the wild-type construct (C) or its proline-to-alanine mutants: P804A (C4), P810A (C10), and P813A (C13).

By performing a GST-pull down using cell lysates of HEK 293T cells transfected with TRPA1 or an in vitro GST-pull down with purified TRPA1 (Fig. 1g, h), it became evident that mutating prolines 810 and 813, but not 804 (outside the binding region, and hence used as an additional control) disrupts TRPA1-FGFR2 binding event.

To map the binding site on TRPA1 and investigate the possibility of its N-terminal ankyrin repeat domain (ARD) binding to FGFR2 proline-rich motif, we truncated the ARD (ΔN) to generate a GFP-tagged ΔN construct. Unfortunately, this construct did not transfect or express well in HEK 293 T cells. To overcome this technical complication, we purified, biotinylated, and immobilized full length TRPA1 and ΔN on streptavidin beads. We then performed an in vitro pull-down assay with purified full length FGFR2, which indicated that truncating TRPA1 ankyrin repeats abrogates TRPA1-FGFR2 binding event (Fig. 1i).

We confirmed the binding between the two purified full-length proteins in vitro by using microscale thermophoresis (MST in Fig. 1j). This yielded an equilibrium dissociation constant (*K*_d_) of 122.8 ± 23.4 nM, which is within the range of *K*_d_ values previously reported for ARD-mediated binding events^21, 22^. On the other hand, a binding curve could not be established with ΔN and full-length FGFR2 (Fig. 1j).

**Fig. 2:**
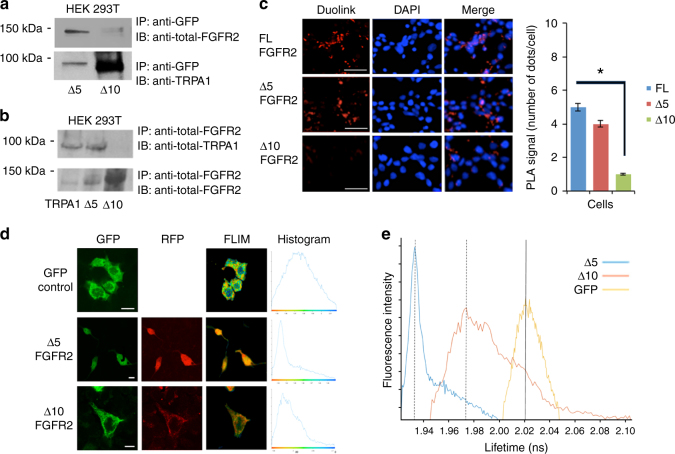
FGFR2 binds to ankyrins 6–10 of TRPA1. **a**, **b** Western blot analysis of an IP with anti-GFP in (**a**) and anti-FGFR2 in (**b**) in HEK 293T cells that were co-transfected with full-length FGFR2 and N-terminal ankyrin repeat truncated TRPA1 (Δ5-GFP or Δ10-GFP) followed by IB with the indicated antibodies. **c** Duolink assay results performed in HEK 293T cells that were co-transfected with FGFR2 and TRPA1 constructs. Scale bar: 50 μm. The bar graph indicates the number of red dots (binding events) in the samples. Error bars, s.d. (*n* = 3 biological replicates each with technical replicates). *P ≤ 0.05 by two-tailed Student’s *t* test. **d**, **e** FLIM experiment in HEK 293T cells co-transfected with FGFR2-RFP and Δ5-GFP or Δ10-GFP. Y-axis dep﻿icts the fluorescence intensity. Scale bar is 10 μm. HEK 293T cells that were transfected with an empty GFP vector (upper panel) were utilized as a control to establish the lifetime of GFP. Histograms (shown on the same scale) in (**e**) indicate lifetime changes in nanoseconds (ns), where a decrease in GFP lifetime (peak shift to the left of the yellow curve) correlates with a potential direct binding event between the two proteins

To identify the binding region on ARD, we truncated the last 5 or the last 10 ankyrin repeats of TRPA1-GFP to generate Δ5 and Δ10 constructs, respectively. Full-length TRPA1, Δ5, or Δ10 were co-transfected with FGFR2 into HEK 293T cells. Since proline-motif interactions prevail under non-stimulated conditions^18, 19^, we serum-starved the cells prior to performing the IP (Fig. 2a, b; the efficiency of transfection is shown in Supplementary Fig. 2h). In these cells, pulling-down with TRPA1 and probing for FGFR2 (Fig. 2a with anti-TRPA1 bands as an IP normalization control) or pulling-down with FGFR2, then probing for TRPA1 (Fig. 2b with anti-FGFR2 bands as an IP normalization control) demonstrates that truncating the last 10, but not the last 5 ankyrin repeats abrogates the binding event. These results were mirrored in the PLA quantification of a Duolink assay (Fig. 2c).

To explore this observation further at a cellular level, we utilized fluorescent lifetime imaging (FLIM) in HEK 293T cells expressing an empty GFP vector (control; upper panel) or co-expressing FGFR2-RFP with Δ5-GFP or Δ10-GFP (Fig. 2d, e). The GFP fluorescence decays were fitted with a single exponential decay law, whereby a reduced fluorescence lifetime (left shift of peaks) indicates fluorescence resonance energy transfer (FRET) due to complex formation. As shown in Fig. 2e, there is a greater FRET efficiency, and thus a higher chance of direct binding of FGFR2 with Δ5 compared to Δ10 (a shift of 0.09 ns vs. 0.052 ns), which corroborates the above IP and Duolink results.

### The binding event activates FGFR2 and inhibits TRPA1

**Fig. 3:**
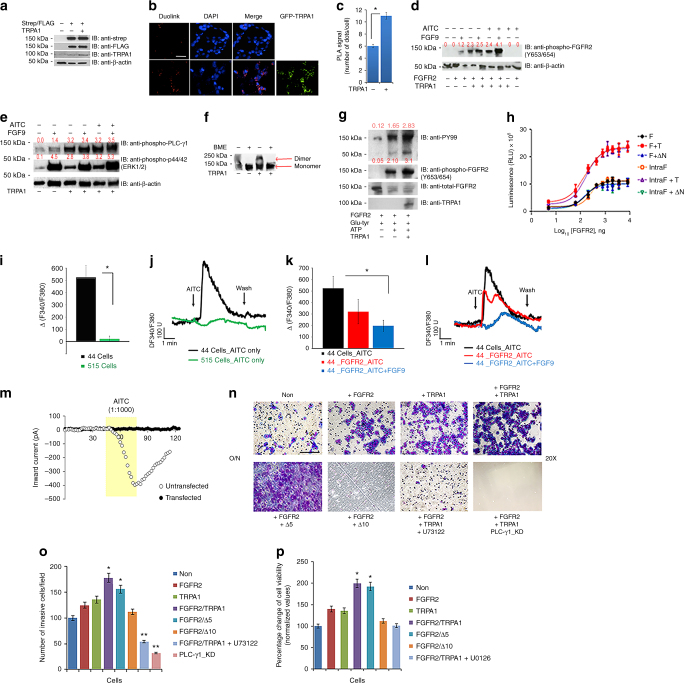
FGFR2-TRPA1 binding event results in FGFR2 activation, but TRPA1 inhibition. **a** Western blot showing the efficiency of transfection of Strep-tagged FGFR2, FLAG-tagged FGFR2, and TRPA1-GFP in HEK 293T cells. **b**, **c** Duolink assay in HEK 293T cells using antibodies targeted against Strep-tagged and FLAG-tagged FGFR2 in the absence (upper panel) or presence (bottom panel) of TRPA1. Scale bar: 50 μm. PLA signals are quantified in (**c**). **d** Western blot analysis of differentially transfected and treated HEK 293T cells. The blot was probed with the indicated antibodies. **e** ﻿Western blot analysis of HEK 293T cells transfected with FGFR2 in all samples but differentially transfected with TRPA1. The blot was probed with the indicated antibodies. **f** Western blot analysis of purified FGFR2 dimerization assay (under non-reducing or reducing conditions) in the presence or absence of purified TRPA1. **g** Western blot analysis of an in vitro receptor kinase assay in the presence or absence of purified TRPA1. **h** Luminescent kinase assay results of purified full length FGFR2 (F) and intracellular cytoplasmic region of FGFR2 (IntraF) in the presence or absence of full-length TRPA1 (T) or ΔN. Intensity of the luminescence signal is measured in relative luminescence units (RLU). **i**–**k** Bar graphs of the averaged calcium imaging results (Δ F340/380) with representative traces of calcium responses. **m** Example inward currents, from a holding potential of −60 mV, evoked in untransfected (○) and FGFR2-transfected HCC-44 cells (●) by the application of AITC (1:1000 in the perfusate). Currents were recorded at 2 s intervals for a control period, during application of AITC and then during washout. **n** Representative images of an invasion assay using differentially transfected HEK 293T that were incubated over night (O/N) in serum starvation media. Lower chamber contains 1% FBS-supplemented media. Images were taken at 20× magnification (scale bar: 100 μm). U73122 is PLC-γ1 inhibitor and PLC-γ1_KD designates HEK 293T cell line with knocked down PLC-γ1. **o** Quantification of the results from (**n**), where the number of invaded cells were counted in 6 different microscopic fields/well. **p** MTT assay results of differentially transfected HEK 293T cells with or without U0126 (MEK inhibitor) treatment. Percentage change in cell viability for each sample with respect to the untransfected control sample (Non) is depicted on the Y-axis. In the above, error bars, s.d. (*n* = 3 biological replicates). *P ≤ 0.05 and **P ≤ 0.01 were determined by two-tailed Student’s *t* test

Next we wanted to investigate whether the TRPA1-FGFR2 interaction is of any regulatory significance. Knowing that FGFR2 dimerization is essential for its activation, we co-transfected Strep-tagged FGFR2 and FLAG-tagged FGFR2 into HEK 293T cells with or without TRPA1-GFP (efficiency of transfection is shown in Fig. 3a) followed by a Duolink assay to test for the co-localization of Strep-tagged FGFR2 and FLAG-tagged FGFR2 according to the experimental procedure described in the literature^23, 24^ (Fig. 3b, c). Our results show that the PLA co-localization signal (number of dots/cells) increases in the presence of TRPA1, suggesting that TRPA1 promotes dimerization of the receptor.

To further assess the significance of TRPA1-mediated FGFR2 activation, we investigated the phosphorylation level of the receptor in HEK 293T cells that were differentially transfected with FGFR2 and/or TRPA1 then left untreated or treated with FGF9 and/or AITC (Fig. 3d). Interestingly, even without stimulation, TRPA1 was capable of inducing FGFR2 phosphorylation on the tyrosine residues (653/654) of its activation loop (Fig. 3d and Supplementary Fig. 3a). To test whether this event is a consequence of TRPA1-mediated calcium influx rather than the outcome of TRPA1-FGFR2 binding event, we treated HEK 293T cells (co-expressing TRPA1 and FGFR2) with AITC with or without TRPA1 antagonist, HC-030031 (Supplementary Fig. 3b). In both cases, FGFR2 phosphorylation was conserved indicating that, indeed, it is the binding event that is responsible for receptor phosphorylation and not TRPA1-activation.

Since phosphorylated FGFR2 is known to be active and capable of recruiting downstream proteins and subsequently activating signaling pathways, we performed a high-throughput reverse phase protein array (RPPA) followed by statistical quantification of the ratio of phosphorylated to total proteins in both HEK 293T cell line and HCC-515 cell line (Supplementary Fig. 3c–e). Based on statistical significance, it appears that PLC-γ1 pathway (implicated in cellular invasion^25, 26^) and the MAPK/ERK cascade (implicated in cellular proliferation^27^) are significantly upregulated in cells expressing both TRPA1 and FGFR2 proteins.

To confirm these results, FGFR2-transfected HEK 293T cells (with/without TRPA1) were serum-starved, left unstimulated or stimulated with FGF9 and/or AITC, and then probed for phosphorylated PLC-γ1 (read out for PLC-γ1 pathway activation) and phosphorylated-p44/42 (ERK1/2, readout for MAPK/ERK pathway activation). The results indicate that both the pathways are activated in cells co-expressing both proteins even in the absence of extracellular stimulation (Fig. 3e; 3rd lane).

It was essential to test whether signaling upregulation is resulting from TRPA1-mediated calcium influx rather than TRPA1-mediated FGFR2 activation. Knowing that pathway activation in HEK 293T cells requires FGFR2 activation (Fig. 3e and Supplementary Fig. 3c–e), we differentially transfected and treated HEK 293T cells in the absence or presence of the calcium-chelating agent, EGTA (Supplementary Fig. 3f). Our results revealed that AITC-mediated TRPA1 activation could not induce ERK signaling in the absence of FGFR2. Additionally, chelating calcium abrogates ERK phosphorylation that is mediated by FGF9-stimulated FGFR2 (2nd and 6th lanes), but not in the presence of AITC-stimulated TRPA1 (4th and 8th lane). This confirmed that: i) AITC alone cannot activate FGFR2 (last lane in Fig. 3d and Supplementary Fig. 3c–e) and ii) TRPA1 seems to trigger downstream signaling pathways by binding and activating FGFR2 independently of the Ca^2+^ influx through the channel.

Next, we wanted to gain insight into the effect of isolated TRPA1 on FGFR2. For that, we utilized purified proteins in an in vitro dimerization assay, whereby we ran a western blot under native vs. reducing conditions using beta mercaptoethanol (βME) in order to disrupt inter-chain disulfide bonds that exist in dimeric FGFR2. On the blot, the potential dimeric band for FGFR2 (higher molecular weight band detected via anti-FGFR2 antibody) appears to be more prominent in the presence of purified TRPA1 (Fig. 3f), which validates the *in situ* results obtained in Fig. 3b. Similarly, to assess the effect of TRPA1-FGFR2 interaction on the in vitro kinase activity of FGFR2, purified FGFR2 with its synthetic substrate (Glu-Tyr) were incubated with ATP in the presence or absence of TRPA1 (Fig. 3g). The results indicate that the kinase activity of the receptor (auto-phosphorylation and substrate phosphorylation) is enhanced in the presence of TRPA1. Additionally, we employed a luminescence kinase assay using purified proteins (linear conversion curve used to extrapolate the luminesce-based kinase activity is shown in Supplementary Fig. 4a). In this case, luminescence signal (proportional to kinase activity) of full-length FGFR2 or the intracellular region of FGFR2 (IntraF; residues 400-821 that encompass FGFR2 kinase domain and the intracellular TRPA1-binding region) is higher in the presence of full length TRPA1 and remains unchanged with ΔN (Fig. 3h).

Taken together, the above data indicate that TRPA1 binds to the C-terminal region of FGFR2 via its ankyrin repeats leading to receptor phosphorylation and subsequent activation of downstream signaling pathways.

We further examined the effect of FGFR2-TRPA1 interaction on TRPA1 activity. Channel activity was assessed, by measuring changes in intracellular calcium, in HCC-515 (expressing both proteins) and HCC-44 cells (lacking FGFR2 expression) treated with AITC (Fig. 3i, j). The results revealed that HCC-44 cells incurred a larger increase of intracellular calcium in response to AITC treatment than HCC-515. Interestingly, the AITC-induced response of HCC-44 cells was significantly reduced when the cells were transfected with FGFR2 (Fig. 3k, l; efficiency of transfection is shown in Supplementary Fig. 4b). Thus, FGFR2 (and specifically its intracellular portion; Supplementary Fig. 4c) has an inhibitory effect on TRPA1 activity that can be inferred from the reduced AITC-mediated calcium influx. This was further validated through patch-clamp experiments performed on HCC-44 cells (Fig. 3m and Supplementary Fig. 4d).

Since TRPA1-FGFR2 binding event can trigger the activation of PLC-γ1 and MAPK/ERK pathways, it was essential to investigate its functional significance. For that, we performed a Boyden chamber invasion assay through a matrigel-coated porous membrane to mimic the extracellular matrix that the invasive cells break down to invade through the pores (Fig. 3n). For the control assay, the chambers with uncoated membrane were utilized (Supplementary Fig. 4e, f). In this case, differentially transfected HEK 293T cells were incubated overnight in the upper chamber in serum starvation media to test their invasive potential into the lower chamber containing 1% serum as a chemoattractant (Fig. 3n). We observed that the cells expressing both TRPA1 and FGFR2 have a higher invasive potential compared to those cells lacking both or one of the two proteins (Fig. 3n; upper panel). In the control assay, the chemotactic potential of the cells was similar (Supplementary Fig. 4e, f).

To determine whether increased invasive phenotype is linked to TRPA1-FGFR2 binding event, and not just TRPA1 and FGFR2 co-expression, HEK 293T cells were co-transfected with FGFR2 and either with Δ5 TRPA1 or Δ10 TRPA1 (Fig. 3n; bottom panel). Our findings demonstrate that truncating the last 10-ankyrin repeats of TRPA1, not only abrogates the binding event, but also diminishes the invasive potential of the cells. To test whether PLC-γ1 pathway is responsible for cellular invasion in this model system, we inhibited the pathway with PLC inhibitor, (U73122) or knocked down PLC-γ1 in HEK 293T cells that co-express FGFR2 and TRPA1 to generate PLC-γ1_KD cell line (Fig. 3n; Bottom panel). In both cases, the invasive potential of the cells was significantly decreased (Fig. 3o). The efficiency of knockdown and the assay quantification results are shown in Supplementary Fig. 4g; upper panel.

Since cellular proliferation is mainly induced by MAPK pathway, we tested the effect of TRPA1-FGFR2 on cellular proliferation using a proliferation/viability MTT assay. For that, HEK 293T cells were differentially transfected with FGFR2 and TRPA1 constructs with or without treatment with MAPK inhibitor, U0126 at 10 µM (efficiency of inhibition is shown in Supplementary Fig. 4g; bottom panel). Notably, deleting the last 10 ankyrin repeats of TRPA1 or treating HEK 293T with U0126 drastically lowered the proliferative potential of the cells compared to that of the untreated cells that have been co-transfected with full-length FGFR2 along with full length TRPA1 or Δ5 TRPA1 (Fig. 3p). This indicates that TRPA1-FGFR2 binding event triggers cellular invasion/proliferation by aberrantly activating FGFR2, independent of extracellular stimulation.

### TRPA1 expression is reduced upon encounter with astrocytes

**Fig. 4:**
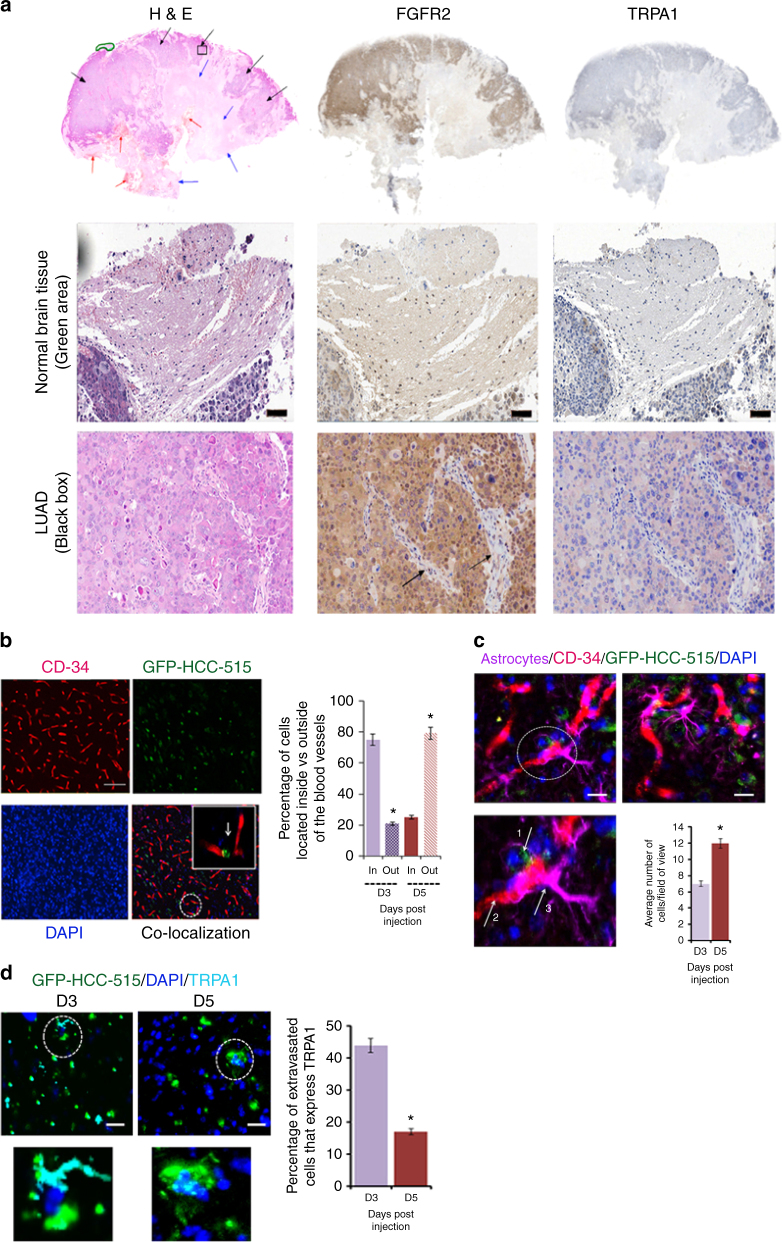
TRPA1 expression level is significantly reduced upon LUAD brain metastasis. **a** IHC and H&E staining of lung cancer brain metastases. Upper panel: Normal brain tissue (green area), tumor tissue (black arrows), necrosis (blue arrows), and haemorrhage (red arrows). Middle panel: Higher magnification of normal brain (scale bar: 100 μm). Bottom panel: Higher magnification of metastatic lung cancer (scale bar: 200 μm) shows FGFR2-positive tumor cells with areas of negatively-stained fibrovascular connective tissue (black arrows). **b** Left panel: Images of IF staining at 20× magnification of mouse brain slices 5 days following injection with GFP-HCC-515. CD-34 (Alexa 555) was used as a blood vessel marker. Scale bars: 20 μm. Right panel: Percentage of cancer cells located inside vs. outside blood vessels 3 days (D3) and 5 days (D5) following injection. Error bars indicate s.d. *P ≤ 0.05. **c** Left panel: Two representative images from D5 (one with an additional zoom-in area). GFAP-positive reactive astrocytes (Alexa 647; pseudo colour: magenta) are shown in the vicinity of extravasating cancer cells (green) as indicated by the white arrows (arrow 1: cancer cell; arrow 2: blood vessel; and arrow 3: astrocyte). Scale bars: 20 μm. Right panel: Bar graph representation of the number of activated astrocytes present in the vicinity of cancer cells 3 days (D3) and 5 days (D5) following injection. Cancer cell-associated astrocytes are quantified within a distance of 150-μm from cancer cells. Error bars indicate s.d. *P ≤ 0.05. **d** Left panel: TRPA1 expression (Alexa 647; pseudo colour: cyan) in cancer cells 3 days (D3) and 5 days (D5) following injection. Scale bars: 20 μm. Right panel: Extravasated cancer cells were counted in 20 randomly chosen fields per brain followed by calculation of TRPA1-positive cells. Error bars indicate s.d. *P ≤ 0.05

Lung cancer is known to metastasize to the brain^1–5^, and for that, we assessed the expression level of both proteins by staining for them in LUAD brain metastases (Fig. 4a). Consistent with published data^28–30^, the green area on the image that marks the normal brain tissue showed weak to negative staining for FGFR2 and TRPA1. Unlike with normal brain tissue, FGFR2 is strongly positive throughout the entire tumor mass, with the fibrovascular supportive stromal tissue and their hyperplastic fibroblasts being weakly positive (see arrows). Notably, most of the tumor cells infiltrating the brain lack TRPA1 expression unlike tumor cells in primary LUAD tissues (Fig. 1a).

To investigate this further, we injected GFP-HCC-515 cells into the carotid artery of a mouse model system and waited for 3 or 5 days (D3 and D5, respectively) as explained in the Methods section and in accordance with the previously established experimental protocol^31^. 5 days following their injection into the artery, LUAD cells were observed in the vicinity of the brain (Supplementary Fig. 4h)

To assess the infiltration of GFP-HCC-515 cells into the brain, floating tissues of the mouse brain (at D3 and D5 post-injection) were stained with CD-34 (blood vessel marker) to assess the extravasation of cancer cells through the blood vessels and into the brain (Fig. 4b; left panel). By quantifying the percentage of cells “in” vs. “out” of the blood vessels based on the localization of GFP-HCC-515 cells and blood vessels (Fig. 4b; right panel), it is evident that the cancer cells have indeed successfully extravasted into the brain niche in a time-dependent manner. This provided us with a model to investigate the mechanism behind TRPA1 depletion in the brain.

Notably, it has been previously shown that in the brain, cancer cells are encountered by reactive astrocytes that modulate the expression levels of cancer cells' proteins^8^. We hypothesized that such intercellular communication could be responsible for the downregulation of TRPA1 observed in the cancer cells invading the brain. To investigate if this is the case, we stained the mouse brain tissues with the astrocyte marker (GFAP; Fig. 4c), where the number of reactive astrocytes (GFAP^+^ cells) concentrated around GFP-HCC-515 cells was drastically higher at D5 vs. D3 post-injection (Fig. 4c; bar graph). This was consistent with the increase in the number of extravasated cancer cells in D5 vs. D3 (Fig. 4b).

To understand the effect of extravasation on TRPA1 expression, we stained for TRPA1 (cyan colour in Fig. 4d) at D3 and D5. The zoomed-in image clearly shows the absence of TRPA1 expression in HCC-515 cells at D5 compared to D3 where the number of TRPA1^+^ cells in D5 is minimal (bar graph). Thus, we hypothesized that astrocytes may be responsible for TRPA1 depletion in cancer cells possibly through intercellular communication as reported with the tumor suppressor protein, phosphatase and tensin homolog deleted on chromosome 10 (PTEN), in breast cancer^8^.

**Fig. 5:**
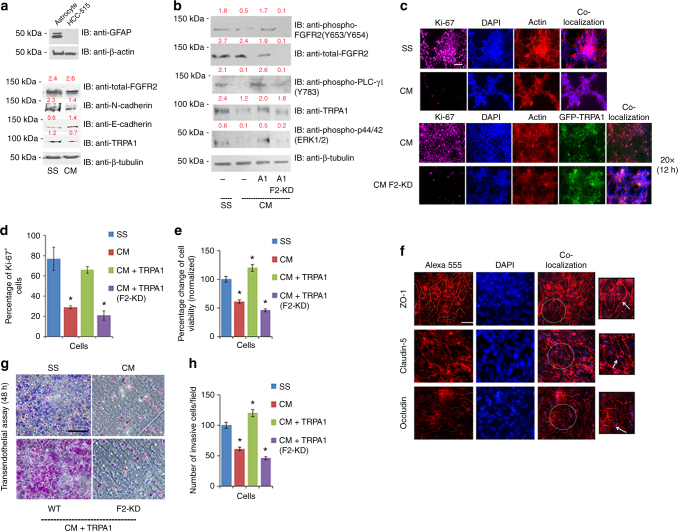
Astrocytes decrease the proliferative and invasive potential of cancer cells by reducing TRPA1 expression level. **a** Upper panel: western blot analysis of GFAP expression level in astrocytes with HCC-515 cells used as a negative control. Bottom panel: western blot analysis of HCC-515 cells that have been serum starved (SS) or treated with conditioned media (CM). The blot was probed with the indicated antibodies. Normalized densitometric values are reported in red above each band throughout the figure and represent the average of three independent experiments. **b** Western blot analysis of HCC-515 cells without TRPA1 transfection (−), with TRPA1 transfection and scrambled shRNA (A1) or with TRPA1 transfection and FGFR2 knockdown (A1/F2_KD). In all cases, blots were probed with the indicated antibodies. **c** IF of HCC-515 cells stained with the proliferation marker, Ki-67. Scale bar: 50 μm. **d** Bar graph quantification of (**c**), where the number of Ki-67^+^ cells were counted under the same magnification in 6 random microscopic fields/sample and averaged. Error bars indicate s.d. (*n* = 3 biological replicates). *P ≤ 0.05 by two-tailed Student’s *t* test. **e** Results of an MTT assay performed overnight and normalized against the values obtained for SS-treated-cells. Error bars indicate s.d. (*n* = 3 biological replicates). *P ≤ 0.05 by two-tailed Student’s *t* test. **f** IF of tight junction proteins (ZO-1, Occludin and Claudin-5 depicted by white arrows in the zoom-in area) in RBE4 cells with different paracellular permeability that were grown in a trans-well chamber. TEER measurement of the integrity of the cellular barrier ranged in value between 150 and 200 ohm cm^2^. Scale bars: 20 μm. **g** Representative images of a transendothelial assay with RBE4 monolayer from (**f**) utilized as a cellular barrier on top of which HCC-515 cells were incubated in SS media or CM media with 1% serum-supplemented media in the lower chamber. Images were taken at 20× magnification. Scale bar: 100 μm. **h** Quantification of the transendothelial assay results, where the number of invaded cells were counted in 8 different microscopic fields. Error bars indicate s.d. (*n* = 3 biological replicates). *P ≤ 0.05 by two-tailed Student’s *t* test

To test this hypothesis further at a cellular level, we isolated astrocytes from the brains of Wistar rats (with GFAP as a marker for isolation efficiency in Fig. 5a; upper panel) and cultured them to prepare astrocyte-conditioned media (CM) as previously described^8^ (see Methods section). Treatment of cancer cells with astrocyte-derived CM is an efficient method to investigate intercellular communication between astrocytes and cancer cells under defined and controlled conditions. This is critical, especially because the growth factor secretion in the brain niche is dynamic and dependent on a myriad of mechanistic and clinical factors^8, 32–34^.

We observed that by treating HCC-515 cells overnight with CM media, TRPA1 expression level was significantly decreased in comparison to the levels in those cells treated with serum starvation (SS) media (Fig. 5a; bottom panel). To test for changes in cellular characteristics, we probed for the expression of E-Cadherin (epithelial marker) and N-cadherin (mesenchymal marker). The results revealed an increase in E-Cadherin level with concomitant decrease in N-Cadherin level, an indicative feature of decreased cellular motility^35^. Notably, the decrease in TRPA1 expression level also resulted in a drastic reduction in the level of phosphorylation of FGFR2, PLC-γ1, and ERK. These effects were rescued upon knocking-in of TRPA1 (A1 cell line), but not when TRPA1 was knocked-in and FGFR2 was knocked-down (A1, F2-KD cell line in Fig. 5b). These findings indicate that astrocytes are inhibiting FGFR2-dependent downstream signaling pathways in HCC-515 cells by decreasing TRPA1 expression level.

To test for functional significance of CM treatment at a cellular level, we stained for Ki-67, which is known to be a proliferation marker^36^ (Fig. 5c, d). Our results demonstrate (based on the percentage of Ki-67^+^ cells) that CM treatment reduced HCC-515 proliferative potential, which was rescued upon knocking-in of TRPA1 in the presence of FGFR2, but not in FGFR2-knockdown cells (bottom panel). Results obtained from this experiment were mirrored in an MTT assay (Fig. 5e).

Furthermore, in order for cancer cells to metastasize to the brain, they need to cross the blood-brain barrier^37, 38^. Thus, for physiological relevance and in order to test the effect of CM on cellular invasion, we performed a transendothelial assay (Fig. 5g, h) with a chemotaxis assay used as a control (Supplementary Fig. 4i, j). For that, rat brain endothelial (RBE4) cells were grown as a monolayer in the upper compartment of a trans-well plate to reach full confluency. Barrier integrity was validated by the high expression of tight junction (TJ) proteins with a typical localization at cellular borders (Fig. 5f). The transendothelial electrical resistance (TEER) of the brain endothelial monolayers, indicating the tightness of TJs, reached 150-200 ohm·cm2 on average, which is within the acceptable range of reported TEER values in RBE4 models^39^. By culturing HCC-515 cells on top of the RBE4-monolayer in SS media (control) or CM media with 1% FBS-supplemented media in the lower chamber, CM treatment significantly decreased the invasive potential of the cells. On the other hand, the chemotactic potential of the control cells cultured in chambers with uncoated membranes was similar (Supplementary Fig. 4i, j). This effect was reversed upon knocking-in of TRPA1 in the presence of FGFR2, but not upon FGFR2 knockdown (Fig. 5g, h). This drop in both invasion and proliferation may likely be due to the decrease in the phosphorylation level of PLC-γ1 and ERK detected in Fig. 5b.

### Astrocytes abrogate TRPA1 expression via exosomal miR-142-3p

**Fig. 6:**
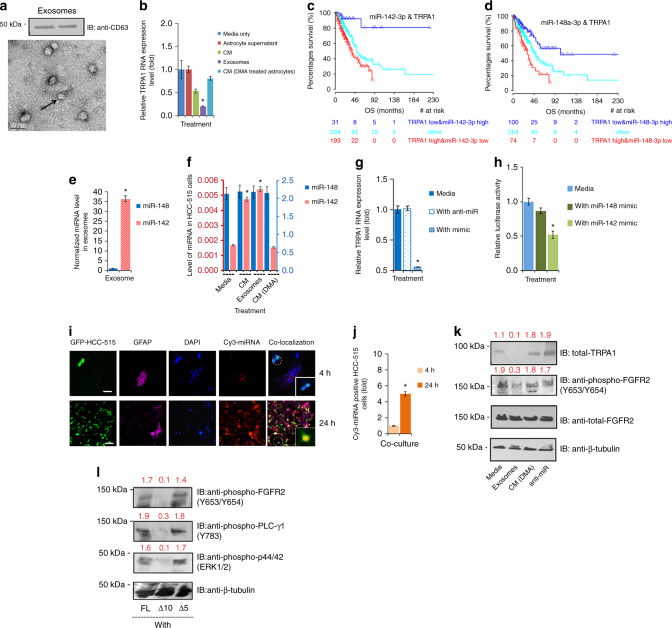
TRPA1-targeting miR-142-3p is intercellularly transferred from astrocytes to HCC-515 cells. **a** TEM image (Scale bar: 50 nm) and western blot analysis (loaded samples are from two separate experiments) of purified exosomes. The blot was probed with CD63 antibody. **b** Bar graph depicting normalized TRPA1 mRNA level in HCC-515 cells treated overnight with SS media (normalization control), astrocyte supernatant, which lacks exosomes, astrocyte-CM, 200 μl of 100X concentrated exosomes and CM from astrocytes that have been treated for 4 h with DMA (inhibitor of exosomal release). **c**, **d** Kaplan–Meier curve depicting the correlation of TRPA1, miR-142-3p, and miR-148-3p with patient survival. The numbers﻿ of patients at risk at different time points are presented at the bottom of the graph﻿. The calculated log-rank test value yielded *P* = 0.00001 (**c**) and *P* = 0.00002 (**d**), respectively. The median of overall survival (OS) in months is shown. **e**, **f** Bar graphs showing the level of miRNA in exosomes and differentially treated HCC-515 cells. **g** Bar graph representation of the changes in TRPA1 mRNA levels in HCC-515 cells following their treatment with SS media only, SS media + anti-miR (miR-142-3p inhibitor) and SS media + miR-142-3p mimic. **h** Bar graph of the 3′-UTR luciferase assay results obtained from HEK 293T cells in the abence or presence of mimics. **i** IF staining images of the intercellular transfer of miR-142-3p, 4 h or 24 h following the co-culture of HCC-515 (expressing an empty GFP vector) with astrocytes that have been loaded with Cy-3-labeled miR142-3p. GFAP (Alexa 647 with magenta as a pseudo-colour) was utilized as an astrocytic marker to distinguish them from cancer cells. Scale bar is 50 μm. **j** Bar graph representation of the results from **i**) counted at 20× magnification in eight randomly selected microscopic fields. **k** Western blot analysis of HCC-515 after variable overnight treatments followed by probing with the indicated antibodies. Normalized densitometric values represent the average of three independent experiments. **l** Western blot analysis of HCC-515 cells 6 h post-transfection with the indicated constructs following their overnight treatment with exosomes. The blot was probed with the indicated antibodies. In the above, error bars, s.d. (*n* = 3 biological replicates). *P ≤ 0.05 and **P ≤ 0.01 by two-tailed Student’s *t* test

Finally, we attempted to elucidate the mechanism modulating TRPA1 expression in LUAD cells upon their encounter with astrocytes. By recognizing the involvement of astrocyte-secreted microRNAs (miRNAs) in depleting the expression of proteins in the brain niche^8, 40, 41^, we postulated that TRPA1 level may be significantly reduced through an miRNA-mediated mechanism. These circulating miRNAs are abundant in the brain niche and are known to be transported as cargo in cell-derived vesicles called exosomes to mediate cellular communication and function^8, 40–43^. Indeed, and via transmission electron microscopy (TEM), we detected the presence of encapsulated particles in CM that varied in size between 30-100 nm, which were positive for the exosomal marker, CD63 (Fig. 6a).

In order to explore the effect of exosomes on the level of TRPA1 mRNA, we treated HCC-515 cells overnight with SS (control 1), CM, concentrated CM-exosomes, residual supernatant from the exosomal extraction process (control 2), and CM collected from dimethyl amiloride (DMA; inhibitor of exosomal release^8, 42, 43^) treated astrocytes (Fig. 6b). Evidently, in the presence of exosomes, TRPA1 in cancer cells was depleted indicating that astrocytes are indeed acting on HCC-515 cells via an exosome-mediated mechanism. Notably, in all these growth conditions, and whenever relevant, exosome-free FBS was utilized instead of regular FBS to ensure the specificity of the experiment.

To establish a better link between astrocyte-derived exosomes and TRPA1 expression level in HCC-515 cells, we investigated all potential TRPA1-targeting miRNAs in LUAD by utilizing The Cancer Genome Atlas (TCGA) database. The main selection criterion used was to identify miRNAs that negatively correlate with TRPA1 expression, but positively correlate with patient overall survival (OS) in LUAD patients. Two miRNAs were identified: miR-148-3p and miR-142-3p (Fig. 6c, d and Supplementary Fig. 5). Notably, and in separate studies, it has been shown that these (species-conserved) miRNAs, can suppress non-small cell lung cancer progression^44, 45^. They have also been shown to exist in the brain microenvironment^46^, yet the link between these miRNAs and LUAD in the brain metastatic niche was never reported.

To determine which of the two miRNAs is implicated in our mechanism, we measured their level in exosomes and uncovered that miR-142-3p, but not miR-148-3p, is abundant (Fig. 6e). On the other hand, in HCC-515 cells, miR-148-3p is more abundant than miR-142-3p with a surge in the level of the latter upon treatment of the cells with purified exosomes (Fig. 6f). Thus, there is a possibility that miR-142-3p is being transferred from astrocytes to HCC-515 cells in astrocyte-derived exosomes leading to TRPA1 depletion.

To investigate the direct effect of miR-142-3p on TRPA1, HCC-515 cells were differentially treated with miR-142 mimic or inhibitor in SS media. As the results indicate, miR-142-3p can indeed regulate the mRNA level of TRPA1 (Fig. 6g). Additionally, we examined the binding of miR-142-3p to the 3′-untranslated region (UTR) of TRPA1, given the requirement for a microRNA to bind to the 3’-UTR of target mRNA in order to deplete protein expression^47^. Data was collected by measuring the luciferase activity of TRPA1 3′-UTR reporter clone in HEK-293T cells. The results show that the signal is significantly reduced in the presence of miR-142-3p mimic, but not miR-148-3p mimic, confirming that miR-142-3p is directly acting on TRPA1 (Fig. 6h).

To investigate the physical transfer of miR-142-3p from astrocytes to HCC-515 cells, Cy-3 labeled miR-142-3p particles (red) were transfected into astrocytes (magenta) that were subsequently co-cultured with GFP-HCC-515 cells (Fig. 6i, j). Our findings show that Cy-3^+^ cancer cells are detected post incubation with astrocytes, signifying the transfer of miR-142-3p from astrocytes to HCC-515 cells.

Finally, to elucidate the link between this intricate mechanism and TRPA1-FGFR2 interaction, we treated HCC-515 cells with SS media, exosomes, CM (DMA), and miR-142-3p inhibitor (Fig. 6k). As shown, treatment with exosomes abrogated TRPA1 expression, which was rescued with CM (DMA) and miR-142-3p inhibitor treatment. These results were mirrored in the level of phosphorylation of FGFR2. To test if such an effect is due to ankyrin-proline binding event, exosome-treated HCC-515 cells were transfected with full-length TRPA1, Δ10, or Δ5 (Fig. 6l). Transfection with full length TRPA1 or Δ5 (both of which lack the 3′-UTR) rescued FGFR2 phosphorylation yet Δ10 (which is incapable of FGFR2 binding and ﻿also lacks the 3′-UTR) failed to do so. This indicates that astrocytes are modulating cellular proliferation and invasion by decreasing TRPA1 expression level and subsequent FGFR2 activity.

## Discussion

TRPA1 mediated protein–protein interactions, their consequent regulatory effects, and roles in LUAD remain largely unknown. Interestingly, the role of TRPA1 as an invasive and prognostic marker in cancer^12, 48^ has recently been reported, reflecting the growing interest in deciphering its oncogenic function. By building on the previously established role of FGFR2 in LUAD^17–19^ and the possibility of ankyrin repeats binding to proline-rich motifs^15^, we investigated the presence of a potential TRPA1-FGFR2 binding event in LUAD and its contribution to the oncogenic process.

Our study clearly indicates that FGFR2 and TRPA1 form a complex, in which the ankyrin repeats of TRPA1 bind to the C-terminal proline-rich region of FGFR2. This interaction inhibits the channel (possibly through structural changes in TRPA-1 conformation), yet acts as a possible conformational scaffold that induces receptor activation independent of extracellular stimulation leading to aberrant proliferation and invasion. This hypothesis, however, opens doors to further future investigations to decipher the structural complexity entailing such an intricate binding event.

Thus, our findings not only identify the regulatory function of TRPA1 ankyrin repeats, but also highlight the importance of ankyrin–proline interactions in tumor progression. This introduces LUAD as a potential TRPA1-modulated channelopathy, with TRPA1 and FGFR2 acting as valuable cancer biomarkers.

In addition, our study brings to light the mechanism by which astrocytes target infiltrating LUAD cells via TRPA1-depleting exosomal miRNA, thereby interrupting the FGFR2-driven oncogenic process at early time points. The trigger for exosomal release by astrocytes, however, remains to be unveiled. Based on previous reports on the crosstalk between cancer cells and astrocytes in the brain, which modulates protein expression in tumour cells^8^, we postulate that the release of chemokines by cancer cells can attract astrocytes and induce the release of exosomal miRNA.

Notably, due to the dynamic nature of the brain microenvironment, the effect of miRNA on TRPA1 might be overcome by cancer cells at later stages^49^. Nonetheless, the herein described miRNA-driven machinery might explain the clinical latency between the appearance of early circulating lung cancer cells and development of brain metastases.

## Methods

### Cell culture

Human Embryonic Kidney cells (HEK 293T) and normal lung fibroblast cell line (CCL-204; CCD-16Lu) were purchased from ATCC®. Normal bronchial epithelial cell line (LIMM-NBE1) was provided by Dr. Philip Egan. Human lung adenocarcinoma cell lines (HCC-44; CSC-C0542) were purchased from Creative Bioarray. Human lung adenocarcinoma cell lines (HCC-515, HCC-827 and NCI-H1793) were provided by Dr. Ignacio I. Wistuba. All cell lines have been tested for mycoplasma contamination and were cultured in Dulbecco’s modified Eagle’s high-glucose medium (DMEM), supplemented with 1% Gibco® Antibiotic-Antimycotic (Life Technologies™) and 10% v/v Gibco® fetal bovine serum (Life Technologies™). All cells were incubated at 37 °C and 5% CO_2_. The immortalized cell line RBE4 (rat brain endothelial cells) was provided by Professor N. Joan Abbott) and used for transendothelial invasion assays^50^. Primary cultures of GFAP^+^ rat astrocytes were obtained from 5-7 days old Wistar rats. According to the protocol^51, 52^, cerebral cortices were placed in fresh PBS free of Ca^2+^ or Mg^2+^ (Sigma Aldrich) and minced in the same buffer containing 0.25 mg/mL trypsin for 15 min at 37 °C following meninges removal. Trypsin was deactivated by 16 μg/mL soybean trypsin inhibitor (type I-S; Sigma Aldrich), 0.5 µg/mL DNase I (EC 3.1.21.1 type II from bovine pancreas, 125 kU/mL, Sigma Aldrich) and 0.3 mM MgSO_4_. The mixture was centrifuged at 400 g for 5 min and the cell pellet was resuspended in 6.8 mL of buffer solution containing 100 µg/mL soy bean trypsin inhibitor, 0.5 µg/mL DNase I and 1.5 mM MgSO_4_. A 10 mL stripette was used to triturate the resuspended cells and the cell suspension was added into 120 mL of Eagle’s minimal essential medium supplemented with 10% fetal calf serum (v/v) and 1% (v/v) penicillin-streptomycin (Invitrogen), then aliquoted into 75 cm^2^ flasks and cultured in 37 °C, 5% CO_2_. Six hours later, the cells were washed and cultured in DMEM at 37 °C, 5% CO_2_ so that the background composition of CM is similar to SS with the only variable being the exosomes that astrocyte have secreted into CM. CM collected from serum-starved astrocytes was prepared by following the established protocol^8, 53, 54^.

### Reagents

FGF9 (catalogue # 273-F9-025) was purchased from R&D Systems. METAFECTENE® PRO (#T040) was purchased from Biontex. TRPA1 antibodies were purchased from Alomone (#ACC-037) and Santa-Cruz (#sc-166469). Total FGFR2 antibody (#sc-122) was purchased from Santa-Cruz. pFGFR2 (#3471), pERK (#4370), Total ERK (#9102) β-actin (#4970), Flag (#8146), Ki-67 (#9449), β-tubulin (#2146), Zona Occludin-1 (#5406), Claudin-5 (#4933), IgG antibody (#2729), anti-GFP (#2956) antibodies along with U0126 (#9903) were purchased from Cell Signalling. U73122 (#1268) was purchased from TOCRIS. NEB 5-alpha competent *E.Coli* (high efficiency) (#C2987H) cells purchased from New England Biolabs, were used for transformation. TRPA1 inhibitor HC030031 (#2896) was purchased from Tocris bioscience. ATP (#9804) and kinase buffer (#9802) were purchased from Cell Signalling. Occludin (H-279) (sc-5562) antibody, validated shRNAs^18, 55^ (sc-29218-V, sc-108080, and sc-29452-V), and DMA (sc-202459) were purchased from Santa Cruz Biotechnology, Inc. Strep antibody (# ab76949) was bought from abcam. CD-63 antibody (#GTX41877) from GeneTex was utilized as an exosomal marker (exosomal proteins were extracted using the Total Exosome RNA and Protein Isolation kit (#4478545, Invitrogen). All antibodies were used in 1:1000 dilutions for western blots.

### Western blotting and pull downs

The cells were left untransfected, or transfected using METAFECTENE® PRO (#T040) from Biontex as previously described^18, 19^. Cells were serum-starved overnight and left un-stimulated or treated with 20 ng/ml FGF9 for 30 min and/or 10 mM AITC for 5 min and/or inhibitors. For extracellular calcium-free condition, the cells were pretreated with calcium-chelating solution (containing 165 mM NaCl, 2.5 mM KCl, 1 mM MgCl_2_, 10 mM Hepes, 10 mM Glucose, and 1 mM EGTA) for 2 h prior to stimulation. After cells lysates were obtained using HEPES lysis buffer, 50 μg of proteins were used for SDS-PAGE and transferred onto PVDF membranes by wet transfer. The membranes were blocked with 5% milk or 3% bovine serum albumin (BSA) and incubated with primary antibodies overnight at 4 °C. After washing, the membranes were incubated with secondary antibody for 40 min at room temperature and washed again prior to visualization with chemiluminescence reagents (Thermo Scientific Pierce® ECL Western Blotting substrate, #32106). For GST-pull down, GST constructs were immobilized on glutathione-sepharose beads, incubated with cell lysates or purified protein followed by western blotting analysis as previously described^18^. For biotin-streptavidin pull down, hTRPA1 and Δ1-688 hTRPA1 proteins were biotinylated with EDC-based carboxyl-biotinylation reagent (EZ-Link™) from ThermoFisher Scientifc. This was followed by pull-down of purified FGFR2 using 0.3 μg/μL of biotinylated bait purified proteins (immobilized on Streptavidin beads) with Pierce™ Biotinylated Protein Interaction Pull-Down Kit (# 21115) followed by western blot analysis. For immunoprecipitation, cell lysates were incubated with Protein A/G Plus Agarose beads (Santa-Cruz, sc-2003) and the experiment was carried out according to the manufacturer’s instructions followed by western blot analysis.

### Calcium imaging

Cells were serum starved prior to incubation with 1 μM Fura-2-AM and 0.01% pluronic acid in EC solution (160 mM NaCl, 2.5 mM KCl, 1 mM MgCl_2_, 2 mM CaCl_2_, 10 mM Hepes, and 10 mM Glucose) at 37 °C for 45 to 60 min. The cells were excited at 340 nm and 380 nm alternatively every 2 s and the calcium response was indicated by the change in fluorescence intensity of Fura-2 at 340/380 nm (F340/F380). Treatment with ionomycin (#I24222, ThermoFisher Scientific) at the end of each experiment was used as a positive control. Every experiment, n, is comprised of the average response of 20–40 cells. Each experiment was performed in triplicates. The imaging was conducted with an inverted Nikon Eclipse TE-2000 microscope, 10X objective, Polychrome V monochromator, IMAGO CCD camera, and TILLvision 4.5 software. In calcium experiments throughout the paper, AITC was used at a concentration of 100 μM.

### Electrophysiology

Fragments of coverslip with attached wild type HCC-44 cells or FGFR2 overexpressing HCC-44 cells were transferred to a continuously perfused (3-5 ml/min) recording chamber mounted on an Olympus (Tokyo, Japan) CK40 inverted microscope. Whole-cell patch clamp recordings were obtained from cells voltage-clamped at -60 mv using an Axopatch 200B amplifier/Digidata 1322 A interface controlled by Clampex 10 software (Molecular Devices, Foster City, CA). The standard perfusate (pH 7.35, 22 ± 1 °C) was composed of 140 mM NaCl, 5 mM KCl, 10 mM HEPES, 1 mM MgCl_2_, and 1.5/2 mM EGTA/CaCl_2_. Patch pipette resistance was 4-6MΩ, series resistance (SR) was compensated (60–80%) after breaking into the cell and periodically monitored throughout the experiment. If SR increased ( > 20%) the experiment was terminated. The intracellular solution (pH 7.35) consisted of 145 mM KCl, 10 mM HEPES, 2 mM MgCl_2_, 10 mM EGTA, 2 mM K_2_ATP, and 0.5 mM GTP. Data were digitized at 5KHz and low-pass filtered at 2.5 KHz. In this experiment, n 3 biological replicates with multiple cells in each.

### MST

The NanoTemper NT.115 machine was used for the thermophoresis experiment as previously described^18^. Purified human TRPA1 with and without the N-terminal ankyrin repeat domain (Δ1-688 hTRPA1) was labeled with Alexafluor 488, and passed through a NAP-5 column (GE Healthcare) to eliminate any unincorporated excess dye. After calculating the concentration of labeled protein, it was used in a constant concentration of 83 nM for the thermophoresis experiment. Titration series were made with 1:1 dilution of purified FGFR2. The experiment was performed in triplicates (repeated 3-8 times) at LED 10–50% and MST power of 40–80%. PBS was supplemented with 0.014% F-14 to solubilize membrane proteins in solutions as previously described^56, 57^. Results of the repetition measurements in the same capillary type with the same IR Laser-power were exported to a txt-file and analyzed with GraphPad Prism software. The baseline was subtracted from each individual experiment and the individual repetition was divided by the amplitude (difference between the unbound and bound state) obtained by fitting the experiment using the Monolith Software. The Y-Axis on the graph represents the “Fraction bound”. Error bars represent the standard deviation of the mean.

### Protein expression and purification

Mammalian expression full-length receptor constructs (FGFR2; HG10824-CF and HG10824-ACR) were purchased from Sino Biological Inc. Strep-tag mammalian plasmid (OGS1159-5UG) was purchased from Sigma. PGEX vector was purchased from GE Healthcare. Full-length GFP-tagged TRPA1 lacking the 3’-UTR (RG219290; coding sequence) was purchased from OriGene. These constructs were used in cloning. Expression and immobilization of GST constructs on glutathione beads was carried out as previously described^18^. Briefly, the relevant constructs were transformed into competent cells that were used to inoculate antibiotic-containing LB media. The culture was grown to an OD600 at 37 °C with constant shaking, then the cells were collected by centrifugation and lysed. Proteins from the cleared extract (filtered supernatant) were eluted, concentrated, and analyzed via SDS-PAGE to assess their purity.

Customized purified full-length FGFR2 was purchased from OriGene and utilized according to manufacturer’s instructions. Purified FGFR2 with His-tag (truncated version IntraF; residues 400-821) was purchased from Sino Biological Inc. The N-terminally His-tagged constructs of human TRPA1 with and without the N-terminal ankyrin repeat domain (Δ1-688 human TRPA1) were overexpressed in the Pichia pastoris expression system and purified as previously described^58^. After Ni-NTA purification, the proteins were desalted and the buffer was changed to PBS supplemented with 0.014% F-14 in a PD-10 column (GE-Healthcare). The proteins were concentrated with Vivaspin 6 with a 100 kDa molecular weight cut-off (Sartorius Stedim Biotech). The protein concentration was determined by measuring the absorbance at 280 nm using a NanoDrop spectrophotometer. Any β-mercaptoethanol used in the purification step was removed before the studies on human TRPA1 and Δ1-688 human TRPA1 were undertaken.

### Kinase assays

Reactions of 20 ng, purified FGFR2 (full-length or cytoplasmic domain (ΔE)) with/without human TRPA1 and/or Δ1-688 human TRPA1 (ΔN), with/without poly(Glu-Tyr) peptide (4:1 or 1:1 molar ratio) were incubated in kinase buffer (#9802 were from Cell Signalling) containing 5 mM ATP,MgCl_2_ and Na_3_VO_4_. Reaction mixtures were incubated at 30 or 37 °C for 20-30 min. Reactions were quenched by adding EDTA (prepared in 10 mM HEPES, pH 7.5) to a final concentration of 25 mM. Samples were then utilized for western blot analysis. The ADP-Glo™ kinase assay (Promega, #V6930) was also used as an additional method to detect FGFR2 kinase activity in 96-well format according to manufacturer’s instructions and as previously described^59^.

### Invasion assay and transendothelial invasion assay

Invasion assays were performed as previously described^18^. In brief, 5 × 10^4^ cells were seeded into the upper chamber of Matrigel Invasion Chambers and Control inserts (Corming, #354480 and #3354578, respectively) with 8 μm porous membrane in serum starvation media. The lower chamber contained 1% FBS media. After 24 h incubation at 37 °C, the upper chambers were cleaned so that there were no cells attached in the inner part, without disturbing the outer side of the porous membrane in which the cells had invaded. After air-drying, the invasive cells were stained (Thermo Scientific quick-diff™ kit, #102164) and counted using the Invitrogen™ EVOS™ FL Cell Imaging System - Thermo Fisher Scientific. The transendothelial assay was adapted from the previously reported methods^60, 61^ The RBE4 monolayer was formed, by seeding 5 × 10^5^ RBE4 in the upper chamber of a Matrigel Invasion Chambers (Corming, #354480), and confirmed by IF-detection of tight junction markers and transendothelial electrical resistance (TEER) measurements using the STX3 electrode and EVOM2 epithelial voltohmmeter (World Precision Instruments, Sarasota, FL, USA). TEER values were measured starting from 24 h after seeding the transwell model with cells, whereby the values obtained with EVOM2 were subtracted from the ohms reading of the blank control (no cells on coated filter) multiplied by membrane surface to get the TEER in ohms.cm2. Readings were carried out in triplicates (n = 3).

### MTT

Serum-starved cells or cells grown in conditioned media were left untreated or treated with 10 µM U0126 for 2 h prior to the MTT assay performed in 12-well plates (3 biological replicates with 3 technical replicates for each). For the assay, 5 mg/ml of MTT (#M5655) was dissolved in RPMI-1640 without phenol red (#. R7509) and the solution was filtered using 0.2 μm filter. Both reagents were purchased from Sigma Aldrich. Briefly, MTT stock solution (5 mg/ml) was added to equal one-tenth the volume of the original culture media and incubated for 3 h and the converted dye was solubilized with 0.1 N HCl in absolute isopropanol followed by measuring the absorbance at a wavelength of 570 nm with background subtraction at 630–690 nm. All assays included blank wells containing medium only, untreated control cells, and test cells of interest. In general, the average values from triplicate readings were determined after subtracting the average value from the blank. The normalization control (such as serum starved cells) represents 100% cell viability. The A570 values of other conditions were divided by the negative control value to determine the percentage change in cell viability for each condition.

### Duolink assay

The Proximity-Ligation Assay (PLA) from Sigma-Aldrich was used in an experiment involving 3 biological replicates each with 3 technical replicates; whereby cells expressing FGFR2 and/or TRPA1 were fixed with 4% paraformaldehyde and then permeabilized with 0.5% NP-40 in PBS. Cells were incubated in blocking buffer (8% FBS, 0.5% NP-40 in TBS) at room temperature for 30 min, and then in primary antibody (1:100, anti-TRPA1 and anti-FGFR2) at 4 °C overnight. Next, the cells were incubated with the PLA probes solution for 1 hour at 37 °C, then with Ligation solution for 30 min at 37 °C and last with the amplification-Polymerase solution for 100 min at 37 °C. The cells were left to air-dry in the dark and image acquisition was carried out following mounting of cells on glass slides, using mounting medium containing DAPI. Number of spots/cell was quantified in multiple (5-8) microscopic fields, averaged, and then used to generate bar graphs.

### Immunofluorescence

Cells cultured on glass coverslips were first washed in PBS, and then fixed in 4% paraformaldehyde for 20 min at room temperature. After washing with PBS, the cells were permeabilized in 0.5% NP-40 in PBS for 5 min and then washed again with PBS. The cells were blocked in blocking buffer containing 8% FBS and 0.5% NP-40 in TBST for 30 min at room temperature. The cells were washed with PBS and then incubated in primary antibody (1:100) at 4 °C overnight. After washing with PBS, the cells were incubated in secondary antibody (1:500) and phalloidin (1:250) for 2 h and then washed with PBS. The cells were mounted on glass slides using mounting media containing DAPI, sealed with nail polish, and then imaged using a confocal microscope or EVOS FL microscope.

### Immunohistochemistry

IHC staining of the TMA (# LUC1021 Pantomics) and brain metastases tissues provided by Leeds Multidisciplinary Research Tissue Bank was performed as previously described^17^ using Access Retrieval Buffer Menarini Diagnostics (Berkshire UK). FGFR2 (1:250) TRPA1 (1:75) were diluted in an Invitrogen antibody diluent, Life Technologies (Paisley UK). Detection was performed with the Menarini X-Cell Plus detection system. Lung cancer patient tissues were provided by the Multidisciplinary Research Tissue Bank that collects and stores the anonymized data and samples from patients who have given full permission for their samples to be used in research.

### FLIM

HEK-293T cells co-transfected with RFP-FGFR2 and Δ5-GFP or Δ10-GFP were cultured on glass coverslips. Serum-starved cells were fixed with 4% w/v paraformaldehyde for 20 min and mounted with mounting buffer. Confocal data were collected with an inverted confocal microscope system (Leica TCS SP2) and two internal photomultiplier tube (PMT) detectors (one displaying the GFP emission and the other one the RFP emission). FLIM data were collected with the same microscope system and a GaAsP hybrid detector (Becker & Hickl HPM-100). The samples were excited with a picosecond diode laser (Hamamatsu PLP-10 470) at 467 nm. The repetition rate of the laser was always 50 MHz, with pulse duration ~90 ps. A 63 × 1.2 NA water-immersion objective was used to acquire the images. A line scan speed of 400 Hz and an image size of 512 × 512 pixels were used for acquiring the confocal images. The FLIM images were acquired with an image size of 256 × 256 pixels. Images decays were analyzed, averaged, and fitted with a single exponential-decay model with SPCImage (Becker & Hickl). The GFP fluorescence lifetime histograms were created in Matlab.

### MicroRNA labeling and luciferase assay

Synthetic miRNA mimics, anti-miR, and negative control miRNAs were purchased from ThermoFisher Scientific, and then labeled when needed and transfected as recommended^8, 62^. Briefly, synthetic miRNA were left unlabeled or labeled with Cy3 using the Silencer® siRNA labeling kit (#AM1632) from Life Technologies. For labeling, miRNA142-3p was incubated for 1 hour at 37 °C in the dark, and then precipitated with ethanol. 100 pmoles of unlabeled/labeled miRNA or anti-miR were transfected into cells grown in 10-cm dishes and cells were incubated for 24 h (in the case of HEK 293T cells) or 48 h in the case of astrocytes that were later co-cultured with LUAD cells at 5:1 ratio and utilized for imaging. For TRPA1 3’-UTR luciferase reporter assay, cells with miRNA mimics (ThermoFisher Scientific) and MISSION(R) 3’UTR Lenti GoClone (TM) TRPA1 (HUTR10124) from Sigma Aldrich were used to measure luciferase reporter activity with Mission Lightswitch Luciferase Assay (#MLS0001) from Sigma Aldrich according to manufacturer’s protocol, with 3 biological replicates each with 3 technical replicates.

### Quantitative PCR qPCRanalysis of mRNA and miRNA levels

Total RNA and miRNA extraction from cells was performed using the mirVana™ miRNA Isolation kit (#AM1560, #AM1561) from Life Technologies. For TRPA1 mRNA measurements, first strand cDNA was synthesized in 20 μl reactions using Superscript II RT (Invitrogen) with 1 μg total RNA and random primers (250 ng). Briefly, the cDNA strand was diluted 1 in 5 with molecular biology grade water prior to use in qPCR reactions. *TRPA1* mRNA expression was assessed using a TaqMan® gene expression assay (Assay ID Hs00175798_m1; Applied Biosystems). qPCR was performed in 20 μl reactions containing 10 μl TaqMan® gene expression master mix (Applied Biosystems), 1 μl TaqMan® gene expression assay, 2 μl of diluted cDNA template, and 7 μl molecular biology grade water. Levels of *TRPA1* expression were normalized to expression of an endogenous control gene, succinate dehydrogenase complex subunit A (SDHA; Assay ID Hs00417200_m1). For miRNA levels, cDNA template for use in qPCR was synthesized using the TaqMan® Advanced miRNA cDNA Synthesis Kit (Applied Biosystems) with 10ng total RNA. Briefly, cDNA was diluted 1 in 10 with molecular biology grade water prior to use in qPCR. Taqman® Advanced miRNA assays were used to assess the levels of hsa-miR-142-3p (Assay ID 477910_mir) and hsa-miR-148a-3p (Assay ID 477814_mir). qPCR was performed in 20 μl reactions containing 10 μl 2X Fast Advanced Master Mix (Advanced Biosystems), 1 μl TaqMan® Advanced miRNA Assay, 5 μl of diluted cDNA template and 4 μl molecular biology grade water. Control miRNA used for normalization was hsa-miR-26a-5p (Assay ID 477995_mir). All reactions were run on a real-time PCR system (Applied Biosystems) and analysed using the comparative Ct (ΔΔCt) method (2^−ΔΔCt^ with logarithm transformation).

### Transmission Electron Microscopy (TEM)

Negative stained grids were prepared using carbon-coated copper grids that had been glow-discharged for 30 s. The sample was blotted and subsequently stained with two drops of 1% Uranyl acetate before drying and imaging. The grids were imaged using a FEI T12 microscope fitted with a 2 K × 2 K. Gatan CCD camera operating at 120 kV was used to examine the samples.

### Staining of floating sections and IF

All procedures were approved by the University of Leeds Animal Welfare & Ethical Review Committee (AWERC), and performed under the approved UK Home Office project license in line with the Animal (Scientific Procedures) Act 1986 and in accordance with the UK National Cancer Research Institute Guidelines for the welfare of animals. The CB17/scid mice used for the study were 6–8 weeks old females bred in-house at St. James’s Biological Services, University of Leeds. Cancer cells (1 × 10^5^) were injected into the left internal carotid artery in a total volume of 50 µL as previously described^31^. Three and 5 days after administration of cancer cells into the carotid artery, the mice were terminally perfused with PBS containing heparin and then with 4% PFA. After the brains were isolated, they were fixed in 4% PFA overnight at 4 °C and then transferred to a 25% sucrose solution (200 mL: 50 g sucrose, 0.5 M NaH_2_PO_4_, and 0.5% Na2HPO4) and incubated at 4 °C until they had sunk (3 days). 35-40 μm sections were sliced from the brains using a cryotome and placed into PBS for immediate staining, or were stored at −20 °C using Walter’s Antifreeze (100 mL: 0.157 g NaH_2_PO_4,_ 0.82 Na_2_HPO_4_ × 2H_2_O, 30 mL Ethyleneglycol, 30 mL glycerol). For staining, floating sections were placed in 12-well plates and washed 3 times with PBS for 10 min. All incubations were performed on the shaker. Sections were incubated with blocking buffer (10% goat serum, 0.3% Triton-X-100 in PBS) for 30–60 min and then incubated with the primary antibody diluted in 0.1–0.2 mL blocking buffer. After washing 3 times with PBS for 10 min, the sections were incubated with secondary antibody in blocking buffer for 2 h at room temperature and then washed again with PBS 3 times. The sections were incubated with DAPI (1:5000 of 10 mg/mL of stock) for 10 min and then washed again with PBS. The sections were treated with ProLong Gold Antifade (Life Technologies, P36930), mounted on glass slides, and left to dry. Images were obtained using an EVOS FL microscope.

### Quantification of extravasation and TRPA1 expression levels

To detect the GFP-tagged tumor cells, floating brain tissues were stained with different combinations of anti-GFP, anti-CD34, anti-TRPA1, and DAPI to visualize localization of cancer cells and blood vessels. The position of extravasated cells inside or outside blood vessels was determined for each detected cell; for this purpose every fourth section throughout the entire mouse brain was analyzed. At both time point (days 3 and 5), four brains were examined (*n* = 4). Sixty randomly chosen events in each mouse were examined per time point. The percentage of intravascular vs. extravascular tumor cells was calculated.

### Quantification of astrocyte association with LUAD cells

Following injection of LUAD cells into the carotid artery, the brain tissues were stained for GFAP to detect astrocytes and co-stained for CD-34 and/or GFP to detect the cancer cells. The number of reactive astrocytes located within a distance of 150 μm from the cancer cells were considered to be cancer-cell-associated astrocytes. The number of reactive astrocytes was counted for 25 randomly chosen events per brain.

### RPPA

RPPA was performed by the Reverse Phase Protein Array Facility within Edinburgh Cancer Research Centre at the Institute of Genetics and Molecular Medicine (IGMM), Western General Hospital Campus, University of Edinburgh. The abundance of total protein and phosphorylated protein epitopes was quantified using the Zeptosens RPPA platform as previously described^63^. In brief, following differential transfections and treatments, the cell culture medium was removed and the cells were lysed with CLB1 buffer (Zeptosens, Bayer) for 30 min. Cell lysates were normalized to a uniform protein concentration with spotting buffer CSBL1 (Zeptosens-Bayer) prior to preparing a final 4-fold concentration series of: 0.2; 0.15; 0.1, and 0.75 mg/ml. The diluted concentration series of each sample were printed onto Zeptosens protein microarray chips (ZeptoChip^TM^, Zeptosens-Bayer) under environmentally controlled conditions (constant 50% humidity and 14 °C temperature) using a noncontact printer (Nanoplotter 2.1e, GeSiM). A single 400 Pico litre droplet of each lysate concentration was deposited onto the Zeptosens chip. A reference grid of AlexaFluor647 conjugate BSA consisting of 4 column × 22 rows was spotted onto each sub-array, each sample concentration series were spotted in between reference columns. After array printing, the arrays were blocked with an aerosol of BSA solution using a custom designed nebulizer device (ZeptoFOG^TM^, Zeptosen-Bayer) for 1 hour. The protein array chips were subsequently washed in double-distilled water and dried prior to performing a dual antibody immunoassay. Following secondary antibody incubation and a final wash step in BSA solution, the immunostained arrays were imaged using the ZeptoREADER^TM^ instrument (Zeptosens-Bayer). For each sub-array, five separate images were acquired using different exposure times ranging from 0.5–10 s. Microarray images representing the longest exposure without saturation of fluorescent signal detection were automatically selected for analysis using the ZeptoView^TM^ 3.1 software. A weighted linear fit through the 4-fold concentration series was used to calculate relative fluorescence intensity (RFI) value for each sample replicate. Local normalization of sample signal to the reference BSA grid was used to compensate for any intra- or inter-array/chip variation. For the heatmap and bar graph generation, all the data points were normalized for protein loading and transformed to a linear value using R console statistical package, Microsoft Excel and ImageJ.

### TCGA

We downloaded publically available (miRNA and mRNA) pre-processed data along with the relevant clinical information and ended up with complete information for 428 patients (please refer to the “Data Availability” section for further details). Survival analyses were performed in R (version 3.0.1) (http:///www.r-project.org/) and the statistical significance was defined as a p-value less than 0.05. First, Univariate Cox proportional hazards model was fitted to evaluate the association between overall survival (OS) and co-variates including TRPA1 and miRNA. This was based on the available expression levels and known important clinical characteristics presented in cbio portal (age at diagnosis, stage, and smoking status). TRPA1 and miRNAs were first evaluated as continuous variables. TRPA1 level and stage were statistically significant factors, from the univariate Cox proportional hazards models. They were included in the multivariable analysis of OS in a model consisting of mRNA, miRNA and stage along with miRNAs found statistically significant at a level of fdr <0.1. Since among miRNAs, miR-142-3p and miR-148-3p showed the highest significance for this study, we continue the description of the methods for these two miRNAs. Patients with high TRPA1 levels and low miRNA expression levels had an increased estimated risk of death when compared to the opposite trend (Supplementary Tables 1, 2 with the raw data available in Supplementary Data 1 Excel Sheet). In order to visualize the survival difference, we used the log-rank test to find the point (cut-off) with the most significant (lowest p-value) split in high vs. low mRNA and miRNA level groups. The Kaplan–Meier plots were generated for these cut-offs. The numbers of patients at risk at different time points are presented at the bottom of the graph. The cut-off to optimally separate the patients in high/low (min p-value) group was 0.34 for TRPA1, 0.72 for miR-142-3p, and 0.28 for miR-148-3p. We then considered adding second expression level. The fixed cut-off for TRPA1 together with the fixed cut-off for miR-142-3p or miR-148-3p splits the cohort in four groups corresponding to low/high TRPA1 and low/high miRNA expression. We contrasted the two groups linked to a negative association: tumors with high levels of TRPA1 and low levels of miRNA vs. tumors with low levels of TRPA1 and high levels of miRNA. The difference in median survival time between the two groups contrasted (38.3 months vs. median not reached for the pair TRPA1 and miR-142-3p and 36 months vs. 104 months for the pair TRPA1 and miR-148-3p) is significantly larger than the difference between the groups classified into high/low based on the expression of TRPA1 or miRNA alone. We also generated survival curves for the 3 groups (the two groups linked to a negative association and the rest of the cases) and verified the largest difference in survival time between the two groups contrasted.

### Data availability

The data supporting the findings of this study are available within the paper and its supplementary information file. MicroRNA (miRNASeq) and mRNA (RNASeqv2) pre-processed data were downloaded from the Cancer Genome Atlas Project (TCGA) publicly available at Broad Institute (http://gdac.broadinstitute.org/) for patients with LUAD. The clinical information was downloaded from cbioPortal (http://www.cbioportal.org/). Source data for the miRNA analysis are provided as Supplementary Data 1. Additional relevant data are available from the authors.

## Electronic supplementary material

Supplementary Information

Description of Additional Supplementary Files

Supplementary Data 1
