# Supplementary material for: TRPA1–FGFR2 binding event is a regulatory oncogenic driver modulated by miRNA-142-3p
Source: Nat Commun. 2017 Oct 16;8:947. doi: 10.1038/s41467-017-00983-w (PMC5643494; doi:10.1038/s41467-017-00983-w)
Supplement: Supplementary file 1 — Supplementary Information [file 41467_2017_983_MOESM1_ESM.pdf]

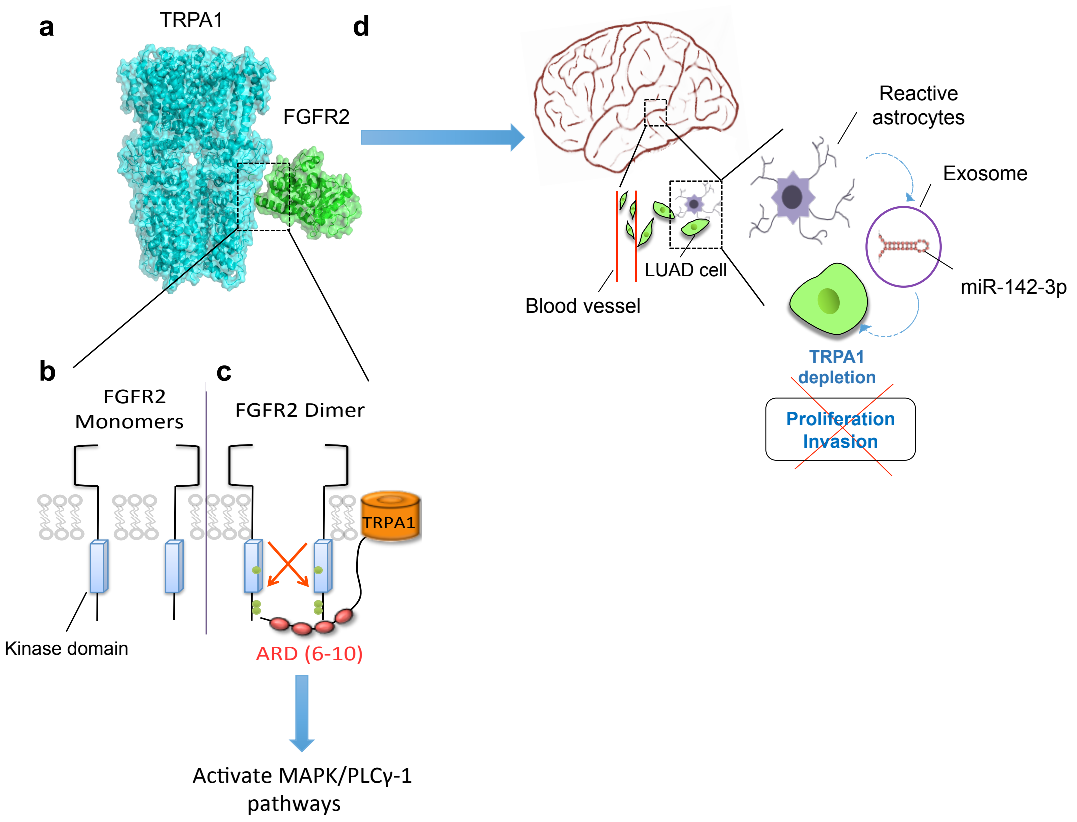

Supplementary Figure 1: Schematic representation of the role of TRPA1-FGFR2 binding event in LUAD disease progression

a, Prediction of the binding event at a structural level where the ankyrin repeats of TRPA1 (blue) form a complex with the C-terminal region of FGFR2 (green). This depiction is based on published 3j9p and 5eg3 crystal structures. **b,** In LUAD cells, FGFR2 monomers cannot activate downstream signalling pathways. **c,** When TRPA1 binds to the C-terminal proline-rich region of FGFR2 via its ankyrin repeats (ARD: ankyrins 6-10), receptor dimerization, phosphorylation (green circles: phosphorylated tyrosine residues) and subsequent activation occur. This binding event inhibits TRPA1 activity but induces FGFR2-mediated aberrant cellular functions, such as proliferation and invasion, independent of extracellular stimulation via MAPK and Plcγ1 pathways. Thus, the potential role of TRPA1 in LUAD is to act as a structural scaffold to mediate FGFR2 activation **d,** TRPA1-FGFR2 binding event induces LUAD metastasis and extravasation into the brain niche. In the brain, LUAD cells will be encountered by reactive astrocytes causing the intercellular transfer of TRPA1-targeting exosomal miR-142-3p from astrocytes to LUAD cells. This event abrogates TRPA1 expression level and consequent FGFR2 activation with its proliferative and invasive effects.

Supplementary Figure 2

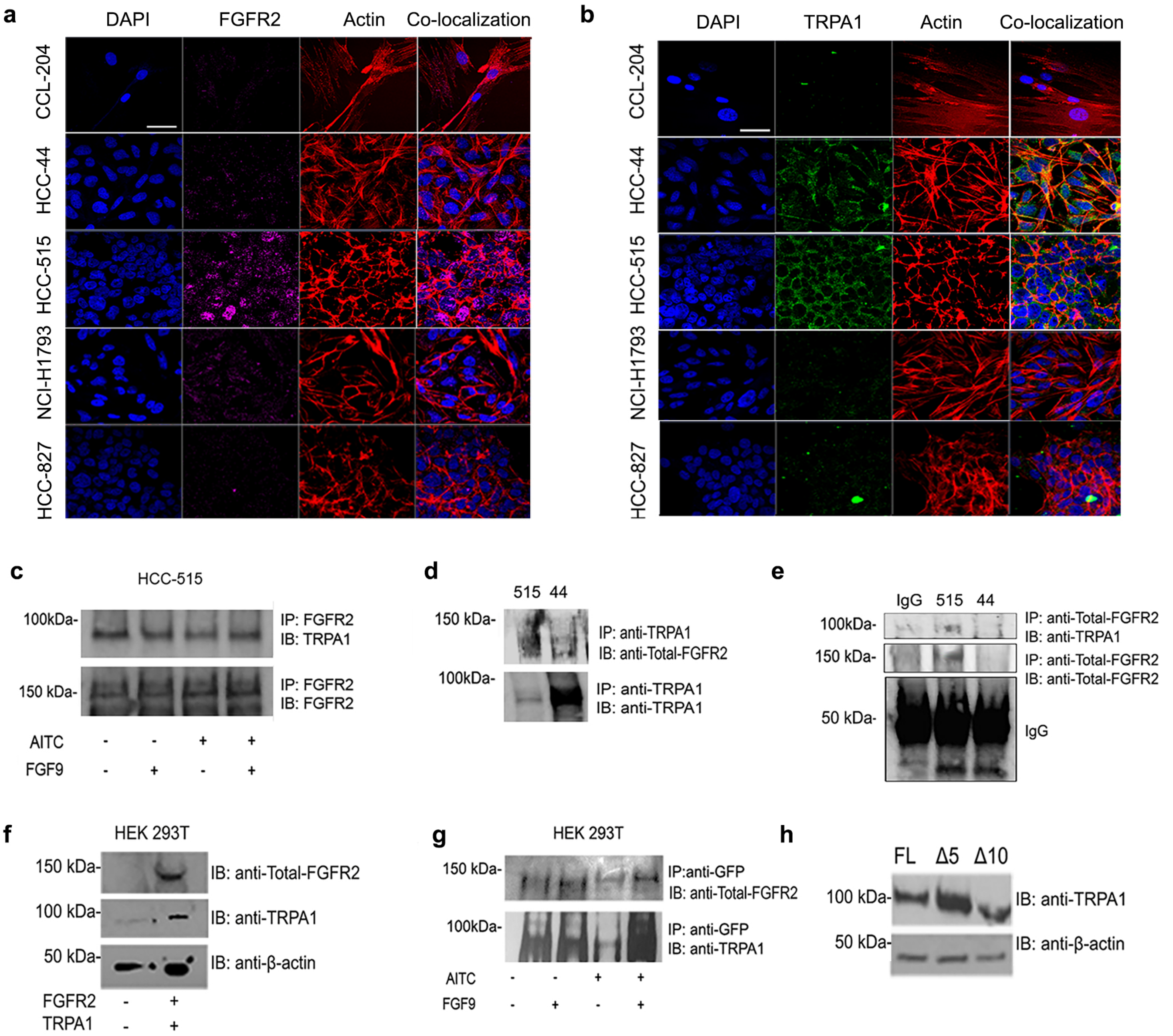

Supplementary Figure 2: TRPA1 and FGFR2 expression levels in LUAD cell lines

a, b, IF images of the expression levels of TRPA1 and FGFR2 in CCL-204, HCC-44, HCC-515, NCI-H1793 and HCC-827 cell lines. Alexa fluor 488 secondary antibody was used against FGFR2 and TRPA1 in separate experiments with magenta as the pseudo-colour for FGFR2. Scale bar: 50 μm. This supports **fig. 1c**. **c,** Western blot analysis of an immunoprecipitation in HCC-515 cells following their differential treatments with AITC and/or FGF9 followed by immunoblotting (IB) with the indicated antibodies. **d,e,** Western blot analysis of an immunoprecipitation in HCC-515 and HCC-44 cell lines followed by immunoblotting (IB) with the indicated antibodies. **f,** Western blot of the efficiency of transfection of full length FGFR2 and full length TRPA1 in HEK 293T cells. The blot was probed with the indicated antibodies. **g,** Western blot analysis of an immunoprecipitation in HEK 293T cells following their differential treatments with AITC and/or FGF9 followed by immunoblotting (IB) with the indicated antibodies. These blots support **fig. 1e**. **h,** Western blot of the efficiency of transfection of full length TRPA1 and its truncation vectors in HEK 293T cells to support **fig. 2**.

Supplementary Figure 3

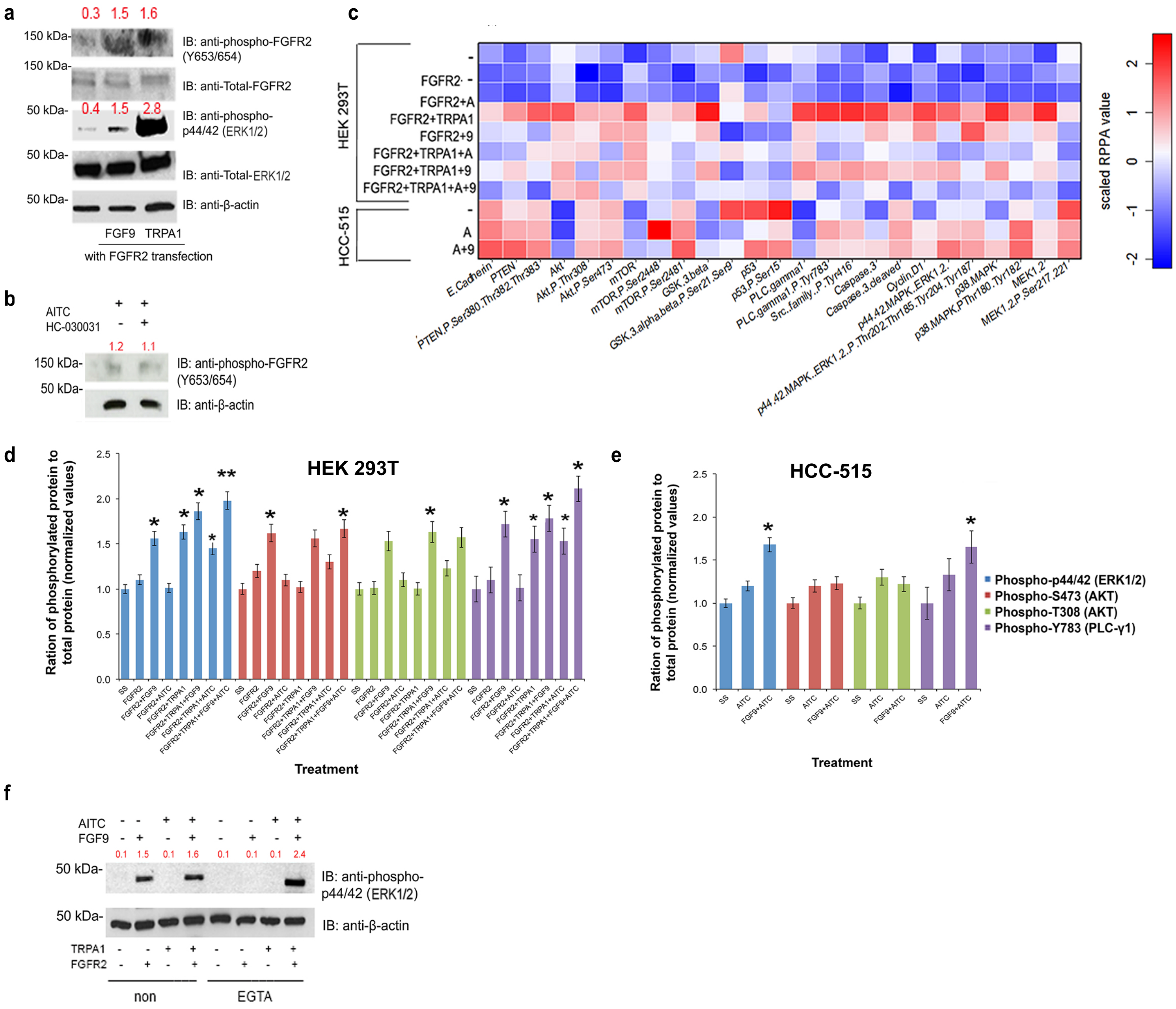

Supplementary Figure 3: TRPA1-FGFR2 regulated downstream signalling pathways.

a, Western blot analysis in HEK 293T cells differentially transfected and treated as indicated on the blot to supplement the results in **fig. 3d,e**. **b,** Western blot of serum starved cells following their treatment with AITC and TRPA1 inhibitor (HC-030031) to support **fig. 3d**. **c,** Heat map of the RPPA data that is based on two independent biological samples that were run in triplicates. TRPA1 and FGFR2 were transiently transfected in HEK 293T cells for this experiment. (-) refers to untransfected/un-treated, (A) is AITC treatment and (9) is FGF9 treatment. **d,e,** Proteins which levels fluctuated upon transfections/treatments were selected and their RPPA values were normalized to actin (loading control) and presented as ratios of phosphorylated proteins to total proteins. Error bars, s.d. (3 runs/sample from 2 biological replicates/sample). *P ≤ 0.05 and **P ≤ 0.01 were determined by two-tailed Student's t test. **f,** Western blot of HEK 293T cells under conditions of variable transfections and treatments with/without a calcium chelator (EGTA). The blot was probed with the indicated antibodies to support **fig. 3e**.

Supplementary Figure 4

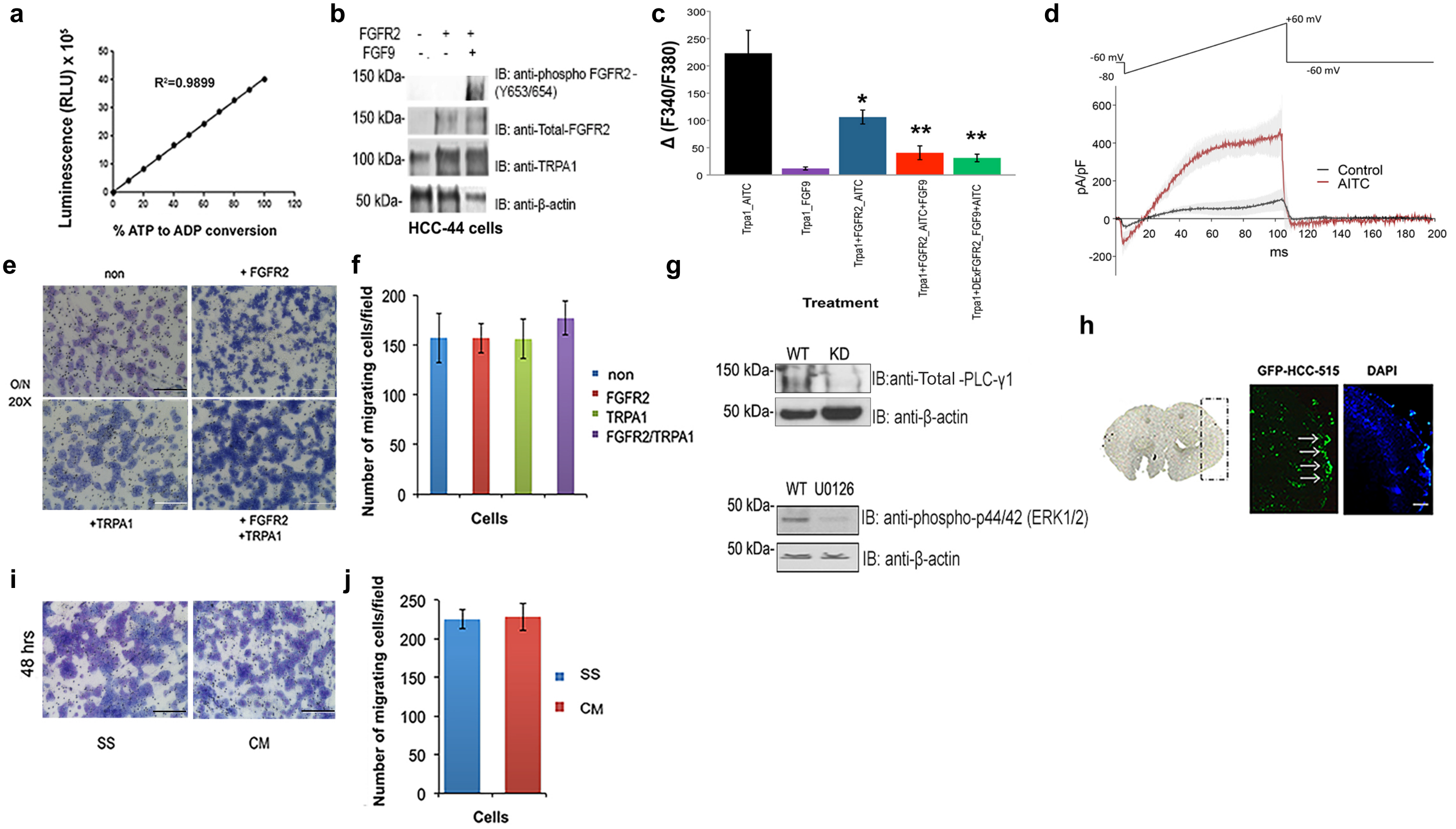

Supplementary Figure 4: Effect of FGFR2-TRPA1 on TRPA1 activity, downstream signalling pathways and cellular functions **a,** ATP to ADP control luminescence experiment indicating the linearity of the conversion mechanism to support the results in **fig. 3h**. n=3 independent experiments. **b,** Western blot of the efficiency of transfection and stimulation in HCC-44 cells to support **fig. 3k, l, m**. **c,** Control calcium imaging experiments in HEK 293T cells differentially transfected with TRPA1, FGFR2, FGFR2 with deleted extracellular region (DExFGFR2) and stimulated with AITC and/or FGF9. Error bars, s.d. (3 runs/sample from 2 biological replicates/sample). *$P \leq 0.05$ and **$P \leq 0.01$ were determined by two-tailed Student's t test. This supports **fig. 3i-l**. **d,** Electrophysiological recordings of HCC-44 cells in the absence (Control) and presence of AITC (1:1000) using a ramp protocol in a whole cell voltage-clamp configuration. Average traces and error bars of currents elicited with a ramp protocol (from -80 mv to +60mV for 100ms) in a whole cell voltage-clamp configuration of control (black trace) and AITC, 1:1000 in the perfusate; red trace. Holding potential (Vh) was -60mV. This supports **fig. 3m. e**, Representative images of a chemotaxis assay where HEK293T cells that have been transfected with the indicated constructs were incubated in the upper chamber in serum starvation media over night (O/N) to evaluate their migratory potential to the lower chamber containing media supplemented with 1% serum. Images were taken at 20X magnification (scale bar: 200 μm). **f,** Quantification of the assay results from **e,)** where the number of cells was counted in 6 different microscopic fields/well. n = 3 replicates. **g, Upper panel:** Western blot analysis of the efficiency of PLCγ1 knockdown (KD) to support **fig. 3n, o**. **Lower panel:** Western blot of HEK293T cells used in **fig. 3p** before and after pre-treatment with MAPK pathway inhibitor, U0126,for 2 hours. Phospho-p44/42 MAP Kinase (Thr202/Tyr204) antibody was used as readout for the efficiency of inhibition. **h, Left panel:** Brain overview section (2X magnification) of a mouse, 5-days following intracarotid injection with GFP-HCC-515 cells. **Right panel:** IF of invading GFP-HCC-515 cells (white arrows) with DAPI nuclear staining. Images were taken at 4X magnification. Scale bar: 200 μm. This supports **fig. 4b,c,d**. **i**, Representative images of a chemotaxis assay with 515 cells that have been incubated in the upper chamber in the indicated media to evaluate their migratory potential to the lower chamber containing media supplemented with 1% serum. Images were taken at 20X magnification (scale bar: 200 μm). **j,** Quantification of the assay results from **e,)** where the number of cells was counted in 6 different microscopic fields/well. n = 3 replicates. This supports **fig. 5g,h.**

Supplementary Figure 5

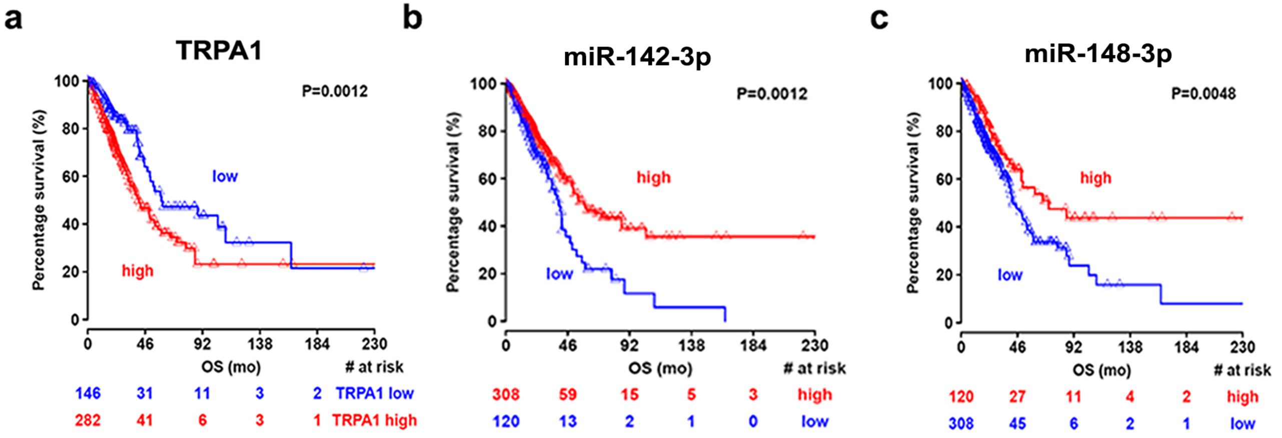

Supplementary Figure 5: TRPA1, miR-142-3p and miR-148-3p correlate with patient survival in LUAD.
a-c, Percentage survival of LUAD patients is presented in a Kaplan–Meier curve based on downloaded and analysed clinical data (TCGA database). The calculated log-rank test value yielded the presented p-values. The median of overall survival (OS) in months is shown. This supplements the data presented in **fig. 6c, d**.

Supplementary Table 1: TRPA1 and miR-142-3p in LUAD patients

	Univariate				Multivariate			
variable	HR	lower.95	upper.95	p-value	HR	lower .95	upper .95	p-value
Age (continuous)	1.20	0.87	1.65	0.268669				
Stage (IV/III vs. I/II)	2.53	1.80	3.55	9.04E-08	2.20	1.55	3.12	1.07E-05
Smoking (ever vs. never)	0.94	0.60	1.49	0.802424				
TRPA1 (continuous)	1.13	1.04	1.24	0.004382	1.13	1.04	1.23	0.005468
miR-142-3p (continuous)	0.87	0.78	0.98	0.026041	0.88	0.78	0.99	0.049219

Supplementary Table 2: TRPA1 and miR-148-3p levels in LUAD patients

	Univariate				Multivariate			
variable	HR	lower.95	upper.95	p-value	HR	lower .95	upper .95	p-value
Age (continuous)	1.20	0.87	1.65	0.268669				
Stage (IV/III vs. I/II)	2.53	1.80	3.55	9.04E-08	2.20	1.55	3.12	1.07E-05
Smoking (ever vs. never)	0.94	0.60	1.49	0.802424				
TRPA1 (continuous)	1.13	1.04	1.24	0.004382	1.12	1.03	1.21	0.01016
miR-148-3p (continuous)	0.84	0.72	0.96	0.01275	0.8	0.68	0.95	0.00935
