# Supplementary material for: TRPA1–FGFR2 binding event is a regulatory oncogenic driver modulated by miRNA-142-3p
Source: Nat Commun. 2017 Oct 16;8:947. doi: 10.1038/s41467-017-00983-w (PMC5643494; doi:10.1038/s41467-017-00983-w)
Supplement: Supplementary file 2 — Description of Additional Supplementary Files [file 41467_2017_983_MOESM2_ESM.pdf]

Description of Additional Supplementary Files

File Name: Supplementary Data 1
Description: An Excel Sheet of the microRNA (miRNASeq) and mRNA (RNASeqv2) pre-processed source data that were downloaded from the Cancer Genome Atlas Project (TCGA) publicly available at Broad Institute (http://gdac.broadinstitute.org/) for patients with lung adenocarcinoma (LUAD). The clinical information in the Excel Sheet was downloaded from cbioPortal (http://www.cbioportal.org/). Data was processed as explained in the "Methods" section of the manuscript (page # 38; TCGA) and utilized to generate the graphs in Figure 6c,d and Supplementary Figure 5.
